# Analysis of the biodegradation of synthetic testosterone and 17α-ethynylestradiol using the edible mushroom *Lentinula edodes*

**DOI:** 10.1007/s13205-018-1458-x

**Published:** 2018-09-28

**Authors:** Bożena Muszyńska, Paweł Żmudzki, Jan Lazur, Katarzyna Kała, Katarzyna Sułkowska-Ziaja, Włodzimierz Opoka

**Affiliations:** 1 Department of Pharmaceutical Botany, Faculty of Pharmacy, Jagiellonian University Medical College, 30-688 Kraków, Poland; 2 Department of Medicinal Chemistry, Faculty of Pharmacy, Jagiellonian University Medical College, 30-688 Kraków, Poland; 3 Department of Inorganic and Analytical Chemistry, Faculty of Pharmacy, Jagiellonian University Medical College, 30-688 Kraków, Poland

**Keywords:** Biodegradation, Edible mushrooms, 17α-Ethynylestradiol, *Lentinula edodes*, Testosterone

## Abstract

The mycelium of *Lentinula edodes* produces enzymes which may degrade xenobiotics including steroid hormones. The aim of the study was to determine whether the mycelium from in vitro cultures of *L. edodes* are able to degrade endocrine disruptors such as testosterone and 17α-ethynylestradiol. To prove the possibility of xenobiotics degradation, cultures of *L. edodes* were cultivated in an Oddoux liquid medium with the addition of synthetic 17α-ethynylestradiol and synthetic testosterone. The endocrine disruptors were extracted from the mycelium and determined qualitatively by RP-HPLC. The degradation products of testosterone and 17α-ethynylestradiol were identified using a UPLC/MS/MS analysis. Undegraded testosterone was determined at the amount of 2.97 mg/g dry weight but only in one of the *L. edodes* extracts from in vitro cultures supplemented with 50 mg of this compound. In turn, 17α-ethynylestradiol was not determined in any samples. Additionally in all extracts, mushroom sterols (ergosterol peroxide and ergosterol) were determined. Their total amounts were significantly lower in samples containing the abovementioned steroids than in extracts from mycelium *L. edodes* without the addition of steroid hormones. The results demonstrated that the mycelium of *L. edodes* can be used in the biodegradation process of a water environment contaminated with endocrine disruptors.

**Electronic supplementary material:**

The online version of this article (10.1007/s13205-018-1458-x) contains supplementary material, which is available to authorized users.

## Introduction

Environmental pollution with xenobiotics (including steroid hormones) has become a serious problem on a global scale. This problem could be solved using white rot fungi (WRF), represented by *Lentinula edodes*. The species has been extensively studied, because its fruiting bodies contain compounds exhibiting anticancer, antioxidant, and antimicrobial effects (Braga 2011; Mleczek et al. 2017; Muszyńska et al. 2017).

Moreover, the mycelium of *L. edodes* produces enzymes with oxidative effects, which may degrade xenobiotics (Kryczyk et al. 2017). White rot mushrooms can transform durable contaminants such as polycyclic aromatic carbohydrates (Lang et al. 1995). These properties can also be used to treat soil contaminated with petroleum. WRF are also efficient in the biodegradation of soils contaminated especially with heavy metals, owing to their capacity to accumulate such metals in the fruiting bodies (Cerniglia et al. 1992; Lang et al. 1995). The efficiency of mushrooms in biodegradation processes is further caused by their rapid growth, production of large amounts of biomass and the widespread occurrence of hyphae in the soil (Ashoka et al. 2002; Wong 2009).

The most important mechanism for the decomposition of xenobiotics by mushroom enzymes is related to the decomposition of lignin. Extracellular enzymes modifying lignin possess low substrate specificity; thus, they can decompose large amounts of highly resistant organic contaminants with a structure similar to that of lignin (Dąbrowska et al. 2018; Mansur et al. 2003). The major enzymes of the lignin degradation system are lignin peroxidase, laccase, manganese-dependent peroxidase, and enzymes producing H_2_O_2_, although not all ligninolytic fungi synthesize them to the same degree (Hofrichter 2002; Kirk and Farrell 1987). Mushroom laccase has been proved able to degrade many pharmaceuticals (for example: naproxen, ketoprofen, diclofenac) (Taheran et al. 2016).

There are numerous sources of environmental contamination by endocrine disruptors. In stock breeding, steroid hormones are used to increase feeding and muscle growth efficiency. The so-called concentrated animal feeding operations (CAFOs) pose a risk to the environment. The feed used in this animal feeding method contains synthetic steroids, which are excreted with their excrements and then leached into water, including groundwater (Anderson et al. 2012). Estrogen has been determined in the excrements and the solid waste of animals and in fertilizers used directly on arable fields (Biswas et al. 2013). Livestock excrements are probably the greatest source of estrogen in the environment. Based on the literature data, pregnant women excrete 260–790 and 280–600 µg/day of estrone and estradiol, respectively. These values are considerably higher than in the case of postmenopausal women treated with hormone replacement therapy (HRT) (Hotchkiss et al. 2008). Additionally, steroid hormones are currently overused by a large group of persons to build their muscle mass. Moreover, hospitals constitute another source of estrogen contamination. Several experiments have corroborated the fact that estrogen, with particularly high levels of estriol, has been determined in samples of hospital sewage (Arnold et al. 2014; Avberšek et al. 2011).

These hormones may contribute to the development of cardiovascular diseases and even cancers (Ibarluzea et al. 2004; Liang and Shan 2013; Moore et al. 2016; Salla et al. 2016). Unfortunately, municipal water treatment plants are not efficient in the removal of steroid hormones from wastewater, thus allowing them to be directly released into the environment (Andaluri et al. 2013; El Osta et al. 2016; Hotchkiss et al. 2008). Removal of endocrine disrupting compounds with WRF was reported (Cruz-Morató et al. 2014).

Therefore, the search for a safe and efficient agent for biodegradation has emerged as an important task. The present study is aimed at the determination (with the use of analytical methods such as RP-HPLC chromatography) of whether *L. edodes*, degrades endocrine disruptors such as 17α-ethynylestradiol and testosterone under in vitro culture conditions.

The degradation products of testosterone and 17α-ethynylestradiol were identified using an UPLC/MS analysis and fragmentation patterns obtained from MS/MS experiments.

## Materials and methods

### Mushroom cultures and reagents

For the experiments, the fruiting bodies of *Lentinula edodes* (Berk.) Pegler of commercial origin, purchased at a local supermarket (2016), were used. The taxonomic identification was based on MycoKey 4.1 software (http://www.mycokey.com) by Muszyńska. Representative samples of the material used for the studies were kept at the Department of Pharmaceutical Botany, Jagiellonian University Medical College (Kraków, Poland).

Some of the young sporocarps of *L. edodes* were used to develop in vitro cultures, in which the obtained mycelium formed the material for further analysis. The explants were degreased with 70% ethanol for 15 s, followed by 0.5 min sterilization in 15% sodium hypochlorite. After repeated washing with sterile redistilled water, the clear fragments of the fruiting bodies were transferred to a solid agar-solidified Oddoux medium (laminar airflow). Cultures from the solid medium were used to establish experimental cultures cultivated on the modified liquid Oddoux medium. The initial inoculum from the solid medium was 0.1 g. The cultures were shaken at the rate of 140 rpm (shaker ALTEL, Poland). They were then incubated at 25 °C ± 2 °C under a photoperiod (10-h light, 900 lx, and 14-h dark). The agitated liquid cultures of *L. edodes* were maintained for 3 weeks and then subcultured.

Standard substances of testosterone, 17α-ethynylestradiol, ergosterol, ergosterol peroxide, glucose, along with maltose extract, casein hydrolysate, B_1_ and B_6_ vitamins, l-asparagine, adenine, yeast extract, and agar were purchased from Sigma-Aldrich (St. Louis, MO, USA). The chemicals MgSO_4_·7H_2_O, NH_4_Cl, KH_2_PO_4_, FeCl_3_, MnSO_4_·H_2_O, ZnSO_4_·7H_2_O, and CaCl_2_·6H_2_O were purchased from PPH Golpharm (Kraków, Poland). HPLC-grade methanol, acetonitrile, and formic acid were purchased from Merck (Darmstadt, Germany). Water (quadruple-distilled) with a conductivity of less than 1 µS cm^−1^ was obtained using an S2-97A2 distillation apparatus (ChemLand, Stargard, Poland).

### Mushroom biomass production and degradation studies

The mycelium of *L. edodes* was passaged in previously prepared flasks, each containing 250 mL of the Oddoux liquid medium. The following amounts of 17α-ethynylestradiol were added to the medium: 100 and 200 µg per 250 mL; those of testosterone were as follows: 25 and 50 mg/250 mL of the medium. Following dissolution in a small volume of ethyl alcohol, the steroids were quantitatively transferred under sterile conditions to flasks containing in vitro cultures of *L. edodes*. Moreover, a control sample was prepared without the addition of steroids. The flasks were placed on an Altel rotary shaker operating at 140 rpm.

After 21 days of the in vitro cultures of *L. edodes*, the biomass was separated from the medium by rinsing with redistilled water. Subsequently, the obtained mycelium and the post-culture media were subjected to lyophilization (Freezone 4.5 lyophilizer by Labconco).

### Extraction, separation and quantification of steroid hormones

Five grams of powdered materials were extracted (mycelium from the in vitro cultures in the media containing endocrine disruptors and the post-culture media) with a mixture of methanol and dichloromethane at a 75:25 (v/v) ratio in an ultrasonic bath at the frequency of 49 kHz for 30 min (Sonic-2, Polsonic). The merged extracts (300 mL) were concentrated to dryness using a rotary vacuum evaporator at 22 °C ± 2 °C and then subjected to RP-HPLC and UPLC/MS/MS analyses. The identity and the amounts of the steroid hormones in the obtained extracts were identified by DAD-HPLC (according to Yuan et al. 2008). The details of the DAD-HPLC analysis, equipment, and conditions were the same as those described by Sułkowska-Ziaja et al. (2015). The quantitative analyses used the standards: testosterone and 17α-ethynylestradiol from Sigma-Aldrich Co. The quantitative analyses were performed using a calibration curve based on the assumption of a linear relationship between the size of the field under the peak and the concentration of the standard substance. Standard substances (by Sigma-Aldrich), testosterone and 17α-ethynylestradiol, were dissolved in a mixture of methanol and dichloromethane (75:25 (v/v)). Solutions with the following concentrations were prepared for testosterone: 1 mg/mL, 0.5 mg/mL, 0.25 mg/mL, 0.125 mg/mL, and 0.0625 mg/L. Further, solutions with the following concentrations were prepared for 17α-ethynylestradiol: 0.25 mg/mL, 0.125 mg/mL, 0.0625 mg/mL, 0.0313 mg/mL, and 0.0156 mg/mL. To determine the possibility of whether the added hormones affect the level of steroids that occur naturally in the fruiting bodies of *L. edodes* (ergosterol, ergosterol peroxide), calibration curves were drawn for these compounds using the following concentrations: 0.5, 0.25, 0.125, 0.0625, and 0.03125 mg/mL.

### UPLC/MS/MS analysis

A Waters ACQUITY^®^ UPLC^®^ from Waters Corporation (Milford, MA, USA) combined with a Waters TQD mass spectrometer were used to perform UPLC–MS/MS analysis. An Acquity UPLC BEH (bridged ethyl hybrid) C_18_ column equipped with an Acquity UPLC BEH C_18_ VanGuard pre-column were used to perform chromatographic separations. The column was maintained under the required conditions. A Waters eλ PDA detector was used to obtain chromatograms. The detailed UPLC–MS/MS analysis was carried out strictly in accordance with the methodology presented by Dąbrowska et al. (2018).

### Statistical analysis

Samples were determined in duplicate. Data were presented as means and standard deviations (SD). The statistical analysis was accomplished using one-way ANOVA followed by a post hoc Tukey test. A *p* value of less than 0.05 was considered significant.

## Results and discussion

Good biomass growth for *L. edodes* was obtained in the liquid cultures on the modified Oddoux medium and on the media containing testosterone and 17α-ethynylestradiol. The dynamics of the mycelium growth in the liquid Oddoux medium did not differ from those registered in earlier studies; however, upon the addition of endocrine disruptors, the growth rate increased twofold (Muszyńska et al. 2013).

The results obtained from the determinations using RP-HPLC for the contents of the steroid compounds in the mycelium extracts from the in vitro cultures in media containing testosterone and 17α-ethynylestradiol and the post-culture media are presented in Table 1.

**Table 1:** Steroid content in samples from mycelial cultures of *Lentinula edodes* grown in Oddoux medium and in media enriched with testosterone or 17α- ethynylestradiol

Mushroom material	Testosterone	17α-Ethynylestradiol	Ergosterol	Ergosterol peroxide
(mg/g)				
Mycelium + testosterone 25 mg	nd	nd	0.54 ± 0.22^a^	0.31 ± 0.07^a^
Medium + testosterone 25 mg	nd	nd	nd	0.86 ± 0.63^b^
Mycelium + testosterone 50 mg	2.97 ± 0.89	nd	0.24 ± 0.09^b^	0.38 ± 0.1
Medium + testosterone 50 mg	nd	nd	nd	0.56 ± 0.17
Mycelium + 17α-ethynylestradiol 100 µg	nd	nd	0.53 ± 0.18^c^	0.29 ± 0.07
Medium + 17α-ethynylestradiol 100 µg	nd	nd	nd	0.7 ± 0.07
Mycelium + 17α-ethynylestradiol 200 µg	nd	nd	0.47 ± 0.38^d^	0.29 ± 0.05
Medium + 17α-ethynylestradiol 200 µg	nd	nd	nd	0.37 ± 0.1
Mycelium from in vitro cultures of *L. edodes* (control)	nd	nd	1.29 ± 0.00^a,b,c,e^	3.56 ± 0.03^a,b^
Medium from in vitro cultures of *L. edodes* (control)	nd	nd	nd	nd

Data presented as mean ± SD (standard deviation). *n* = 6 repetitions. Tukey–Kramer test was used to reveal the differences between paired groups of phenolic compounds in rows, the same letters are marked for the content whose differences are statistically significant (for *p* values < 0.05) (GraphPad InStat)

*nd* not determined

The concentrations of 17α-ethynylestradiol and testosterone used in the experiments as additives to the mycelial cultures of *L. edodes* typically corresponded to the doses of these hormones normally used by patients (17α-ethynylestradiol: 100 and 200 µg/250 mL; testosterone: 25 and 50 mg/250 mL of the medium). The presented experiment demonstrated the capacity of in vitro cultures of *L. edodes* to degrade 17α-ethynylestradiol and testosterone. None of the extracts from the mycelium from in vitro cultures in media containing 17α-ethynylestradiol or the post-culture media were found to contain 17α-ethynylestradiol. In the case of the extracts from the mycelium and the post-culture media of *L. edodes* from in vitro cultures incubated with testosterone, total biodegradation occurred solely in those cultures in medium containing 25 mg/250 mL of the hormone. The testosterone content was determined to be, on average, 2.97 mg/g d.w., and only in the mycelium from the in vitro cultures of *L. edodes* enriched with the addition of testosterone was the content 50 mg/250 mL of the medium.

The experiments demonstrated that the mycelium of *L. edodes* resulted in the decomposition of the endocrine disruptors: 17α-ethynylestradiol and testosterone.

The ligninolytic enzymes produced by *L. edodes* exhibited oxidative properties and were responsible for the degradation of the tested hormones in the in vitro cultures. The degradation of 17α-ethynylestradiol was observed in the experiments of Eldridge et al. (2017) and Riggins and Gregory (2015). The supplementation of *L. edodes* in the in vitro cultures with compounds increasing the production of laccase increased the rate of 17α-ethynylestradiol degradation. Moreover, the main product determined in both the mass spectra was hydroxylated 17α-ethynylestradiol. The results obtained by the abovementioned authors confirmed that laccase contributes to the degradation of 17α-ethynylestradiol (Eldridge et al. 2017; Riggins and Gregory 2015).

Furthermore, the current study included an analysis of the biodegradation products obtained in the cases of endocrine disruptors added to the mycelium.

An analysis of the extracts of the pure medium and of the mushroom mycelium without the added testosterone and 17α-ethynylestradiol showed no peaks on the UV chromatogram; thus, all of the compounds observed on the chromatograms of the extracts of the mushroom materials with the added steroids were most probably products of their biodegradation.

The degradation products of testosterone and 17α-ethynylestradiol were identified using an UPLC/MS analysis and fragmentation patterns obtained from MS/MS experiments. The proposed structures of the degradation products for testosterone are shown in Table 2 and for 17α-ethynylestradiol in Table 3. The proposed fragmentation patterns of testosterone and 17α-ethynylestradiol are presented schematically in Table 4.

**Table 2:**
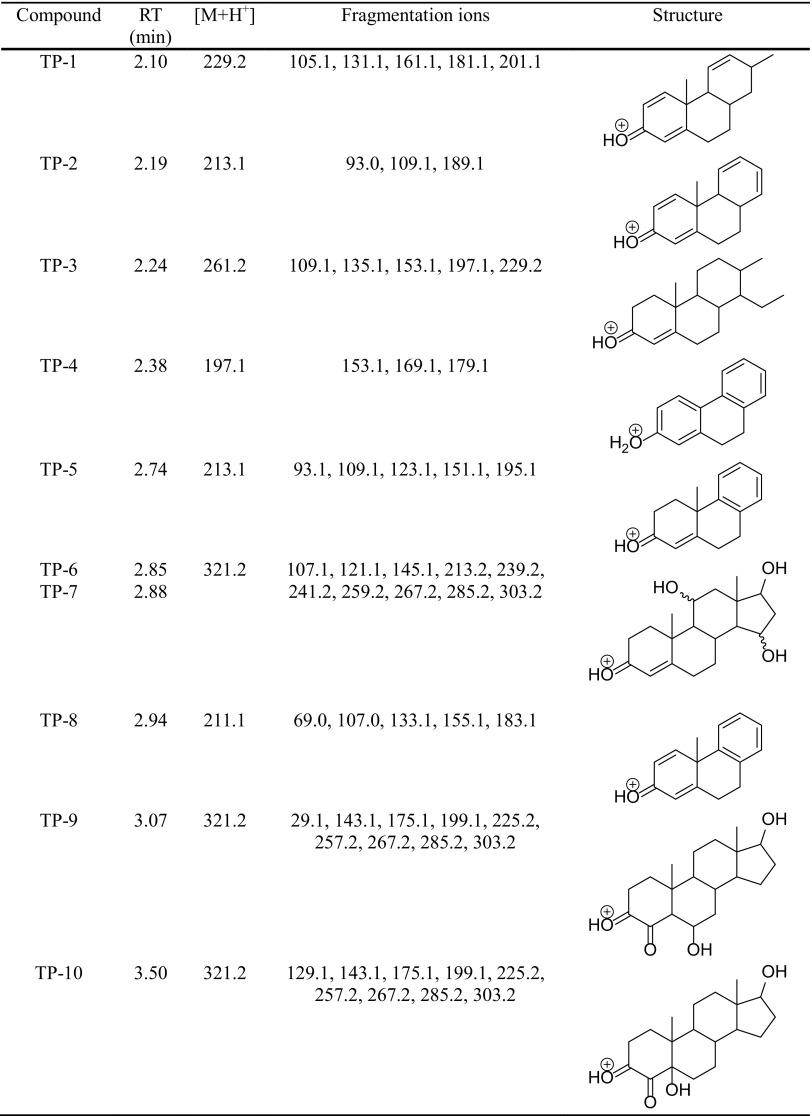

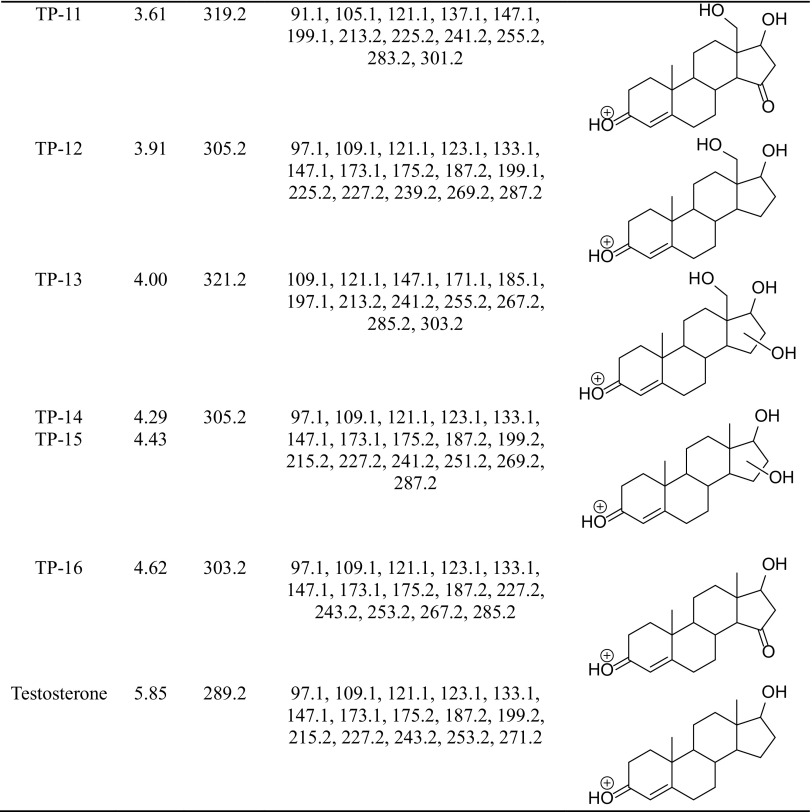
Proposed structures of the biodegradation products of testosterone

**Table 3:**
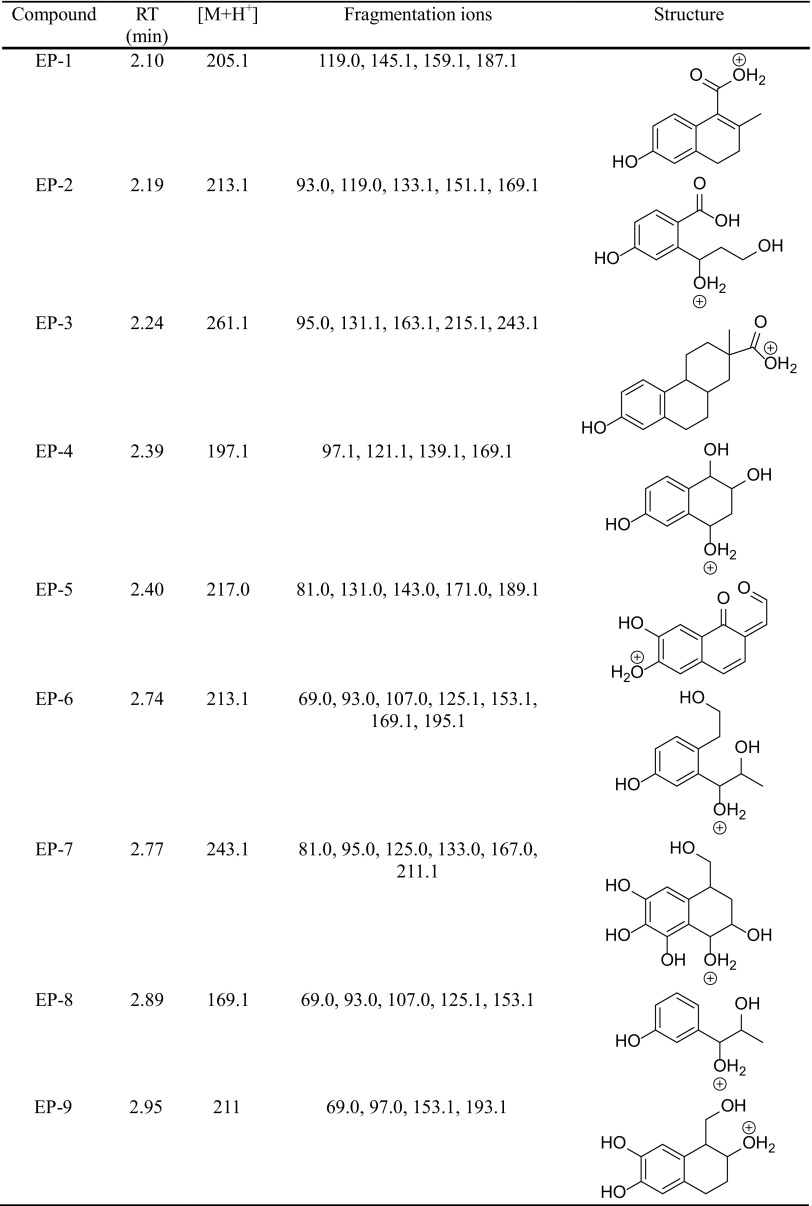
Proposed structures of the biodegradation products of 17α-ethynylestradiol

**Table 4:**
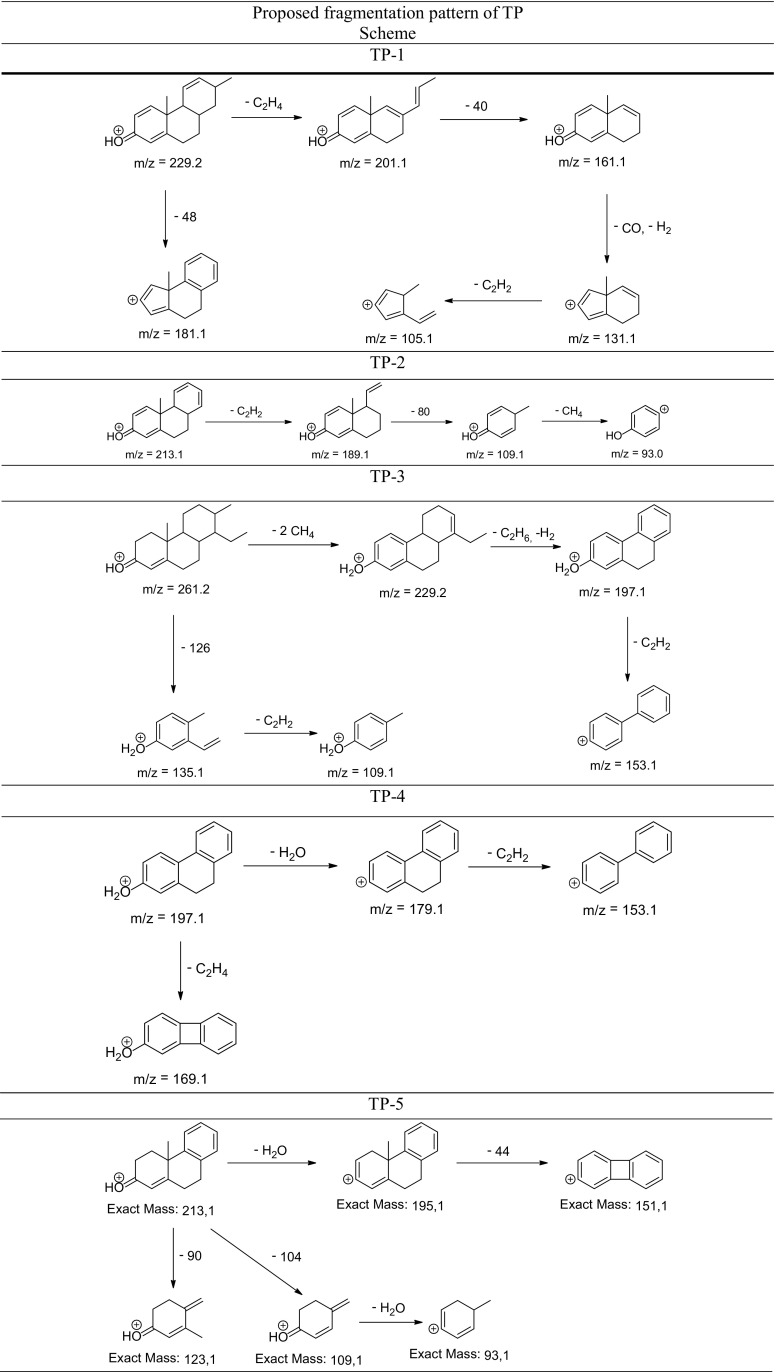

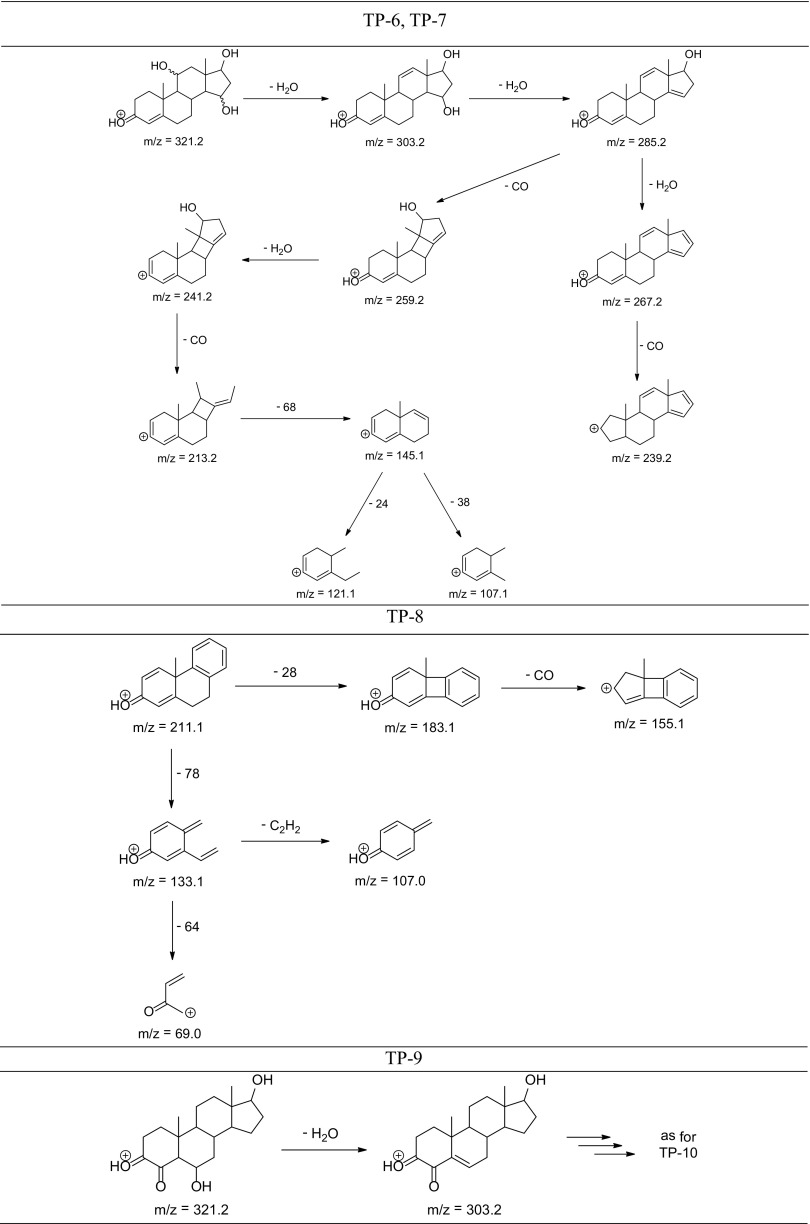

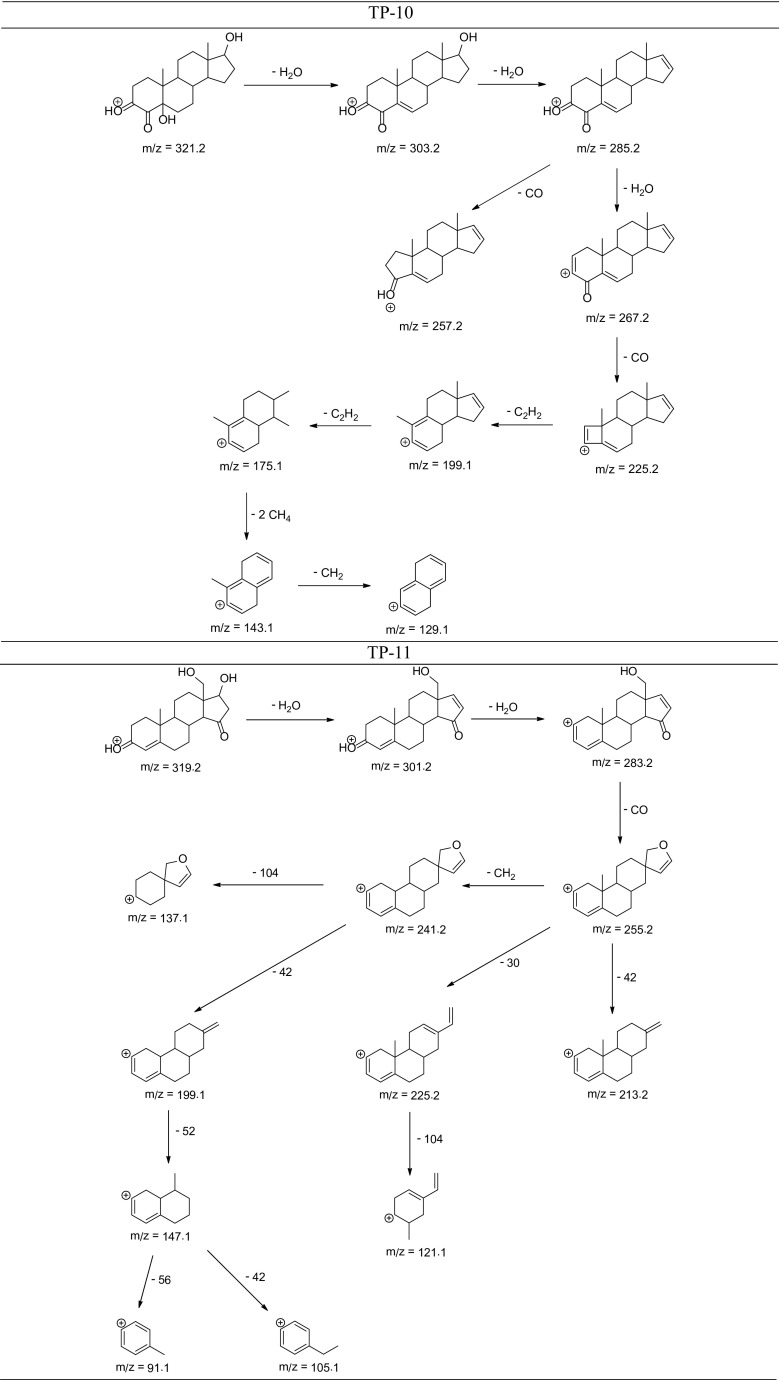

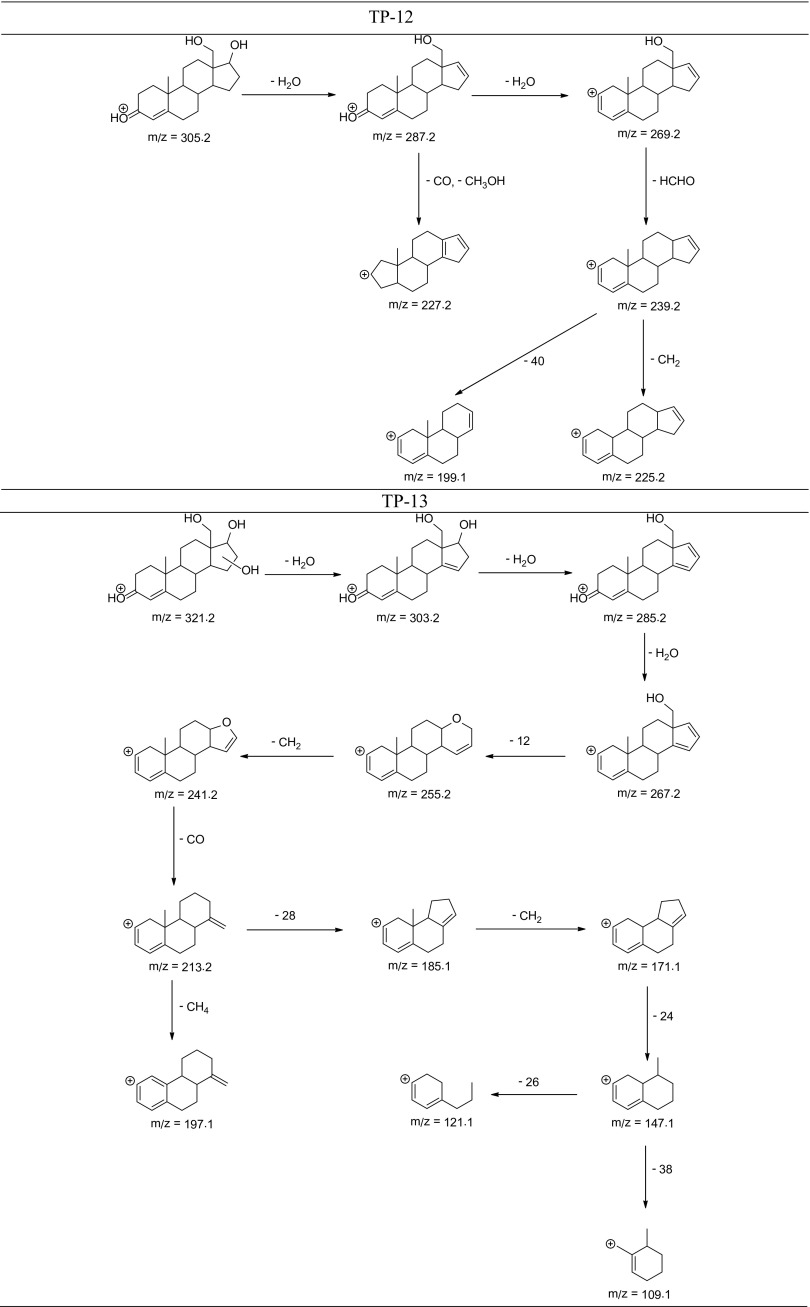

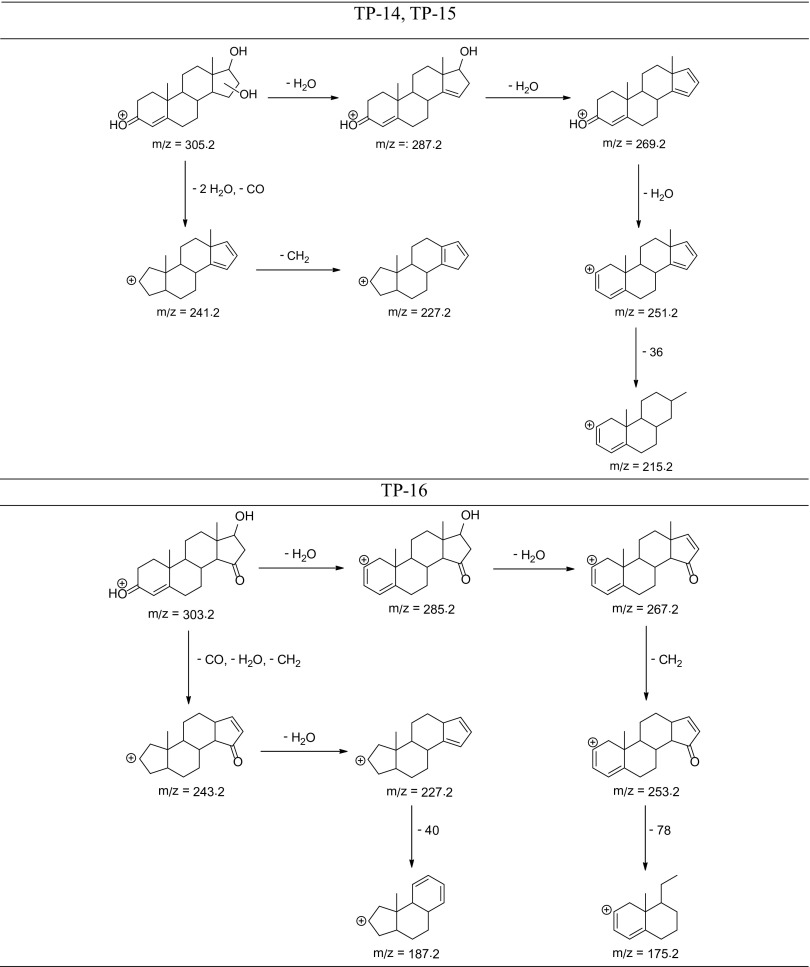

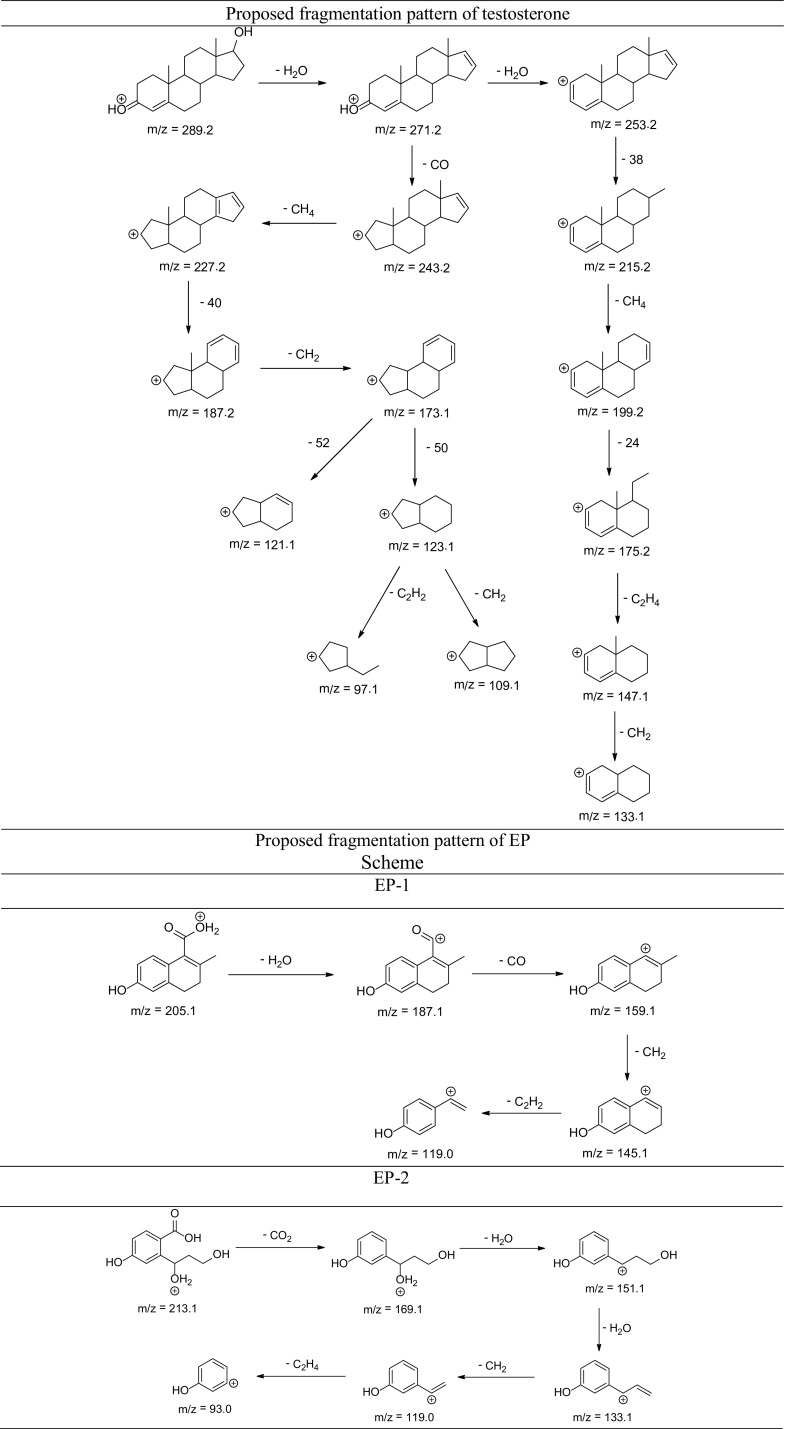

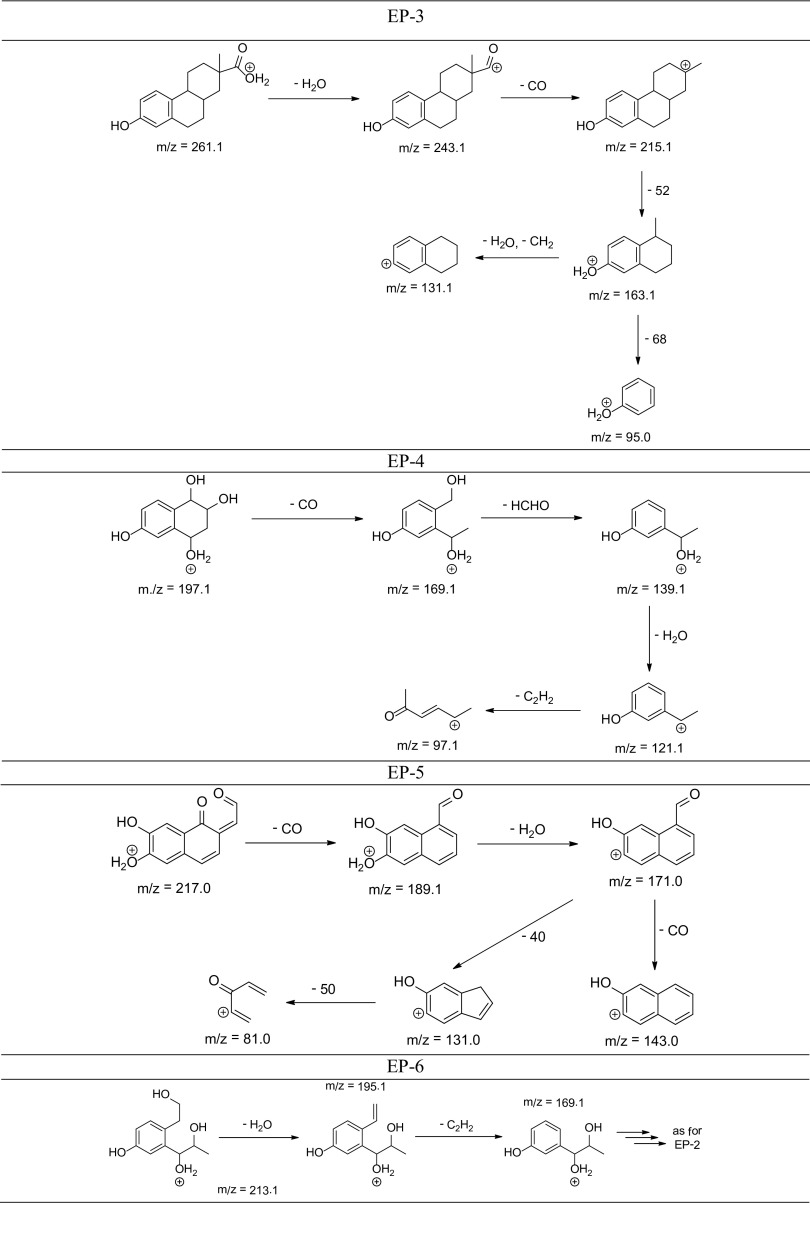
Proposed fragmentation pattern of testosterone and 17α-ethynylestradiol

The degradation process was found to primarily affect rings C and D of the steroids, leading to their oxidation and cleavage. Additionally, the oxidation of ring B was observed, and to a lesser extent, that of ring A. The degradation process was considerably more effective for 17α-ethynylestradiol, for which only a small amount of compounds were found with the remaining A and B rings and only one compound possessing rings A–C. In the case of testosterone, the degradation process was less effective and all of the compounds observed in the extracts from the cultures retained rings A–D or A–C.

No major differences were observed in the degradation products ratios between the samples from the cultures with different amounts of the steroids.

The experiments further determined the level of ergosterol and ergosterol peroxide. None of the tested steroid compounds were found in the extracts from the medium used for in vitro cultures of *L. edodes* (control). Ergosterol was determined solely in mycelium. Ergosterol and ergosterol peroxide occur in a majority of representatives of Basidiomycota species. The mean ergosterol content in the fruiting bodies of genus *Lactarius* is 2.69–3.00 mg/g d.w. and in *Cantharellus* 3.04–3.77 mg/g d.w. In turn in mycelial cultures of *Sarcodon imbricatus* amounts of ergosterol and ergosterol peroxide were 1.97 mg/g d.w. and 2.00 mg/g d.w., respectively (Sułkowska-Ziaja et al. 2016). It was proven that these compounds are essential for the normal development of hyphae of higher fungi while ergosterol is the main part of fungal cell membranes. Although it occurs in a majority of Basidiomycota species, the highest content of this compound was noted in saprophytic fungi (Brennan et al. 1975). We found that the extract from the *L. edodes* mycelium contained a higher amount (3.65 mg/g d.w.) of ergosterol peroxide than that of ergosterol (1.29 mg/g d.w.). Further, the addition of testosterone and 17α-ethynylestradiol to the culture medium resulted in a reduction of the ergosterol and ergosterol peroxide production by the mycelium of the studied species. The ergosterol content in the extracts from the *L. edodes* mycelium from cultures without the addition of endocrine disruptors was, on average, 65.5% higher than in the extracts from the mycelium of the experimental cultures. Moreover, the mycelium extracts contained an average of 91.08% more ergosterol peroxide than the extracts from the *L. edodes* mycelium from those cultures enriched with testosterone and 17α-ethynylestradiol.

The experiment showed that the addition of synthetic steroids influenced the inhibition of the synthesis of endogenous metabolites, such as ergosterol and ergosterol peroxide.

## Conclusions

Endocrine disruptors such as 17α-ethynylestradiol and testosterone, even at concentrations of nanograms per liter, disturb the functioning of the hormone system of vertebrates; therefore, their biodegradation is necessary. The search for new technologies for treating water containing compounds that disturb the action of the hormone system is an important task and, as demonstrated by the present study, the *L. edodes* mycelium may be of use for this purpose.

Steroids in the environment can potentially create persistent toxins. The above-discussed preliminary tests have shown that the examined *L. edodes* mycelia require only a short time period to remove testosterone and 17α-ethynylestradiol from the medium, and thus, mycodegradation can be used as an alternative to the other methods of the biodegradation of steroids compounds contaminating the environment, particularly water.

## Electronic supplementary material

Below is the link to the electronic supplementary material.

Supplementary material 1 (DOCX 6423 KB)
