# Supplementary material for: Analysis of the biodegradation of synthetic testosterone and 17α-ethynylestradiol using the edible mushroom Lentinula edodes
Source: 3 Biotech. 2018 Sep 28;8(10):424. doi: 10.1007/s13205-018-1458-x (PMC6162194; doi:10.1007/s13205-018-1458-x)
Supplement: Supplementary file 1 — Supplementary material 1 (DOCX 6423 KB) [file 13205_2018_1458_MOESM1_ESM.docx]

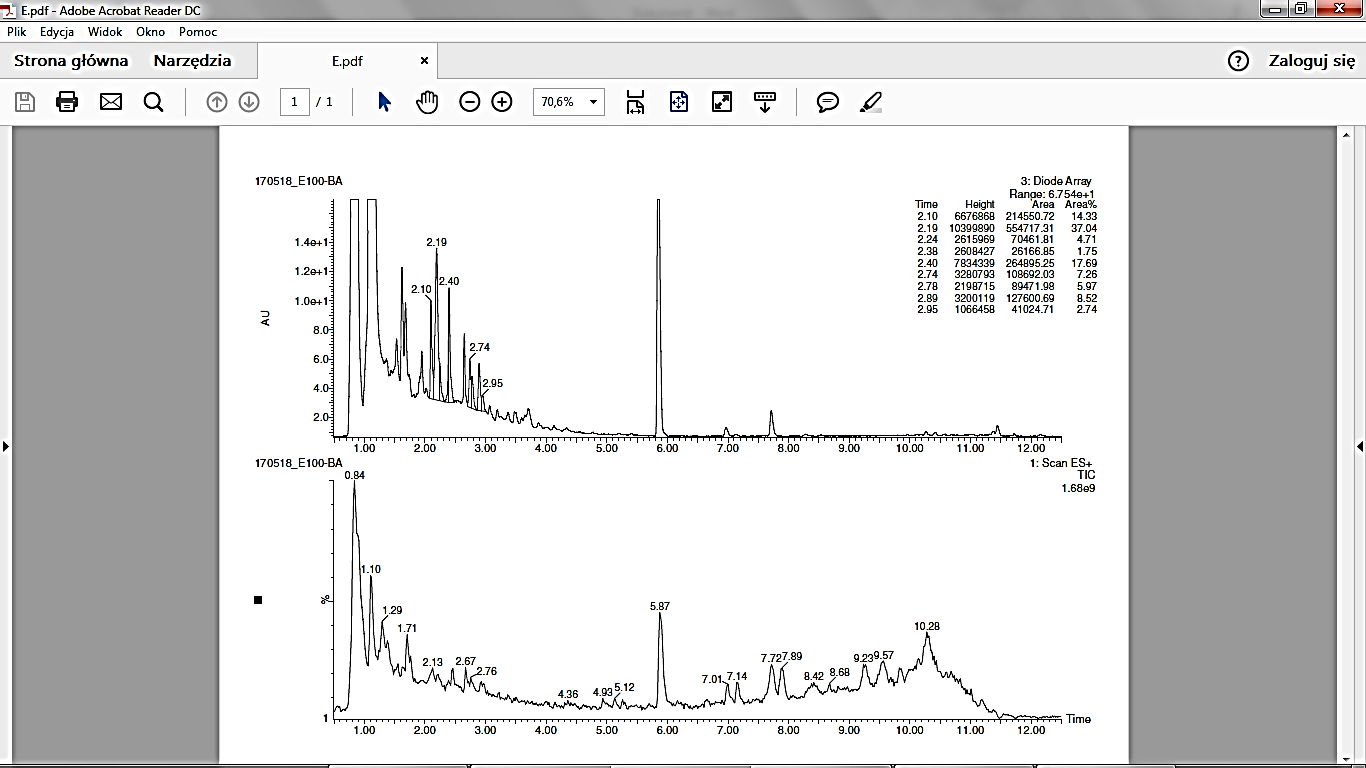


Fig. 1. Chromatogram of mycelium extract after addition of 17α-ethynylestradiol


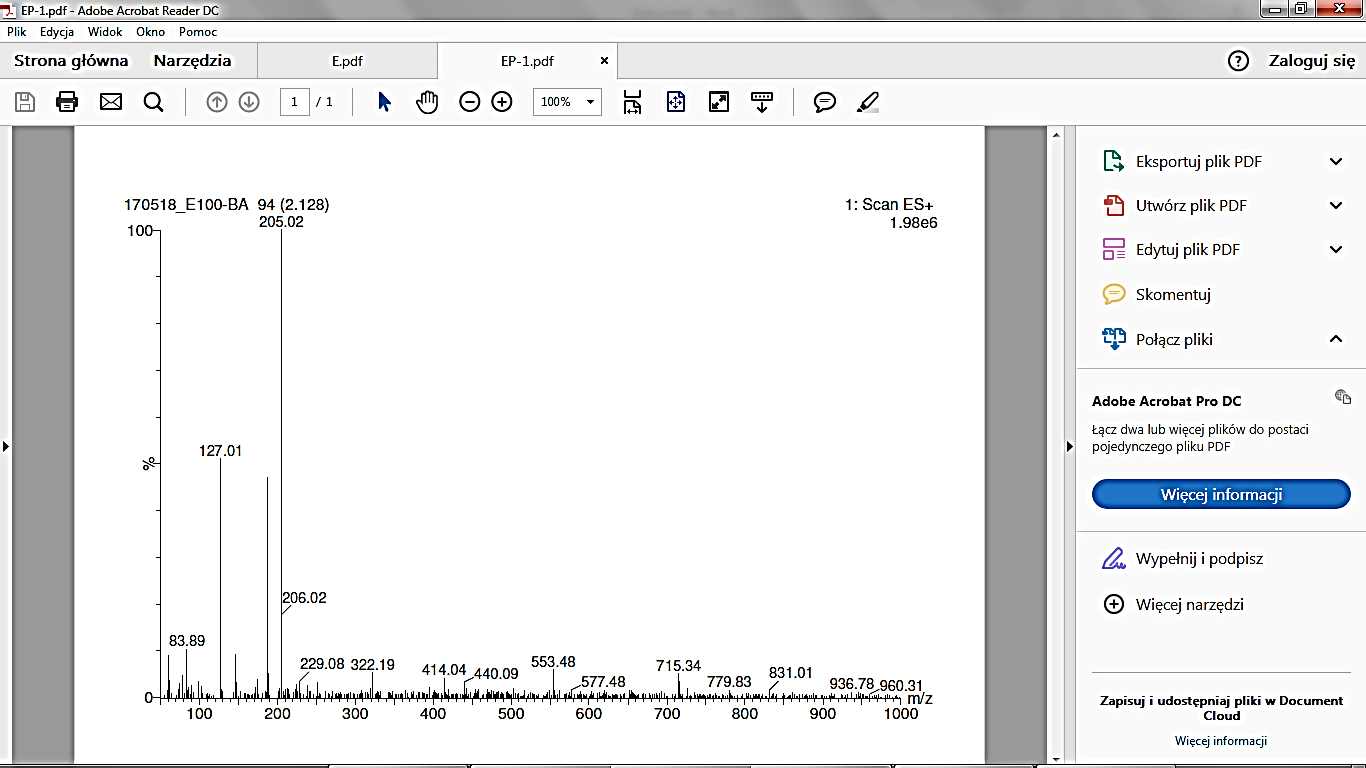


Fig. 2. MS spectrum of EP-1


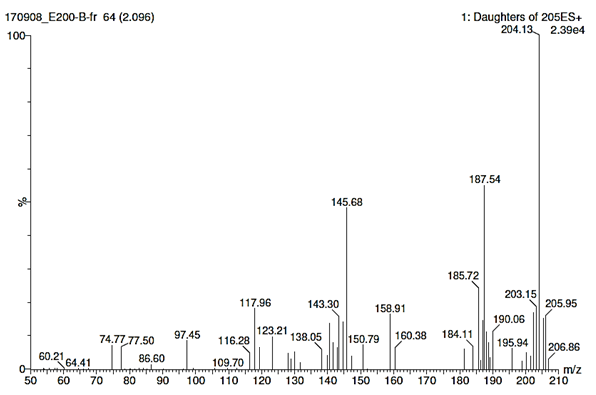


Fig. 3. MS/MS spectrum of EP-1


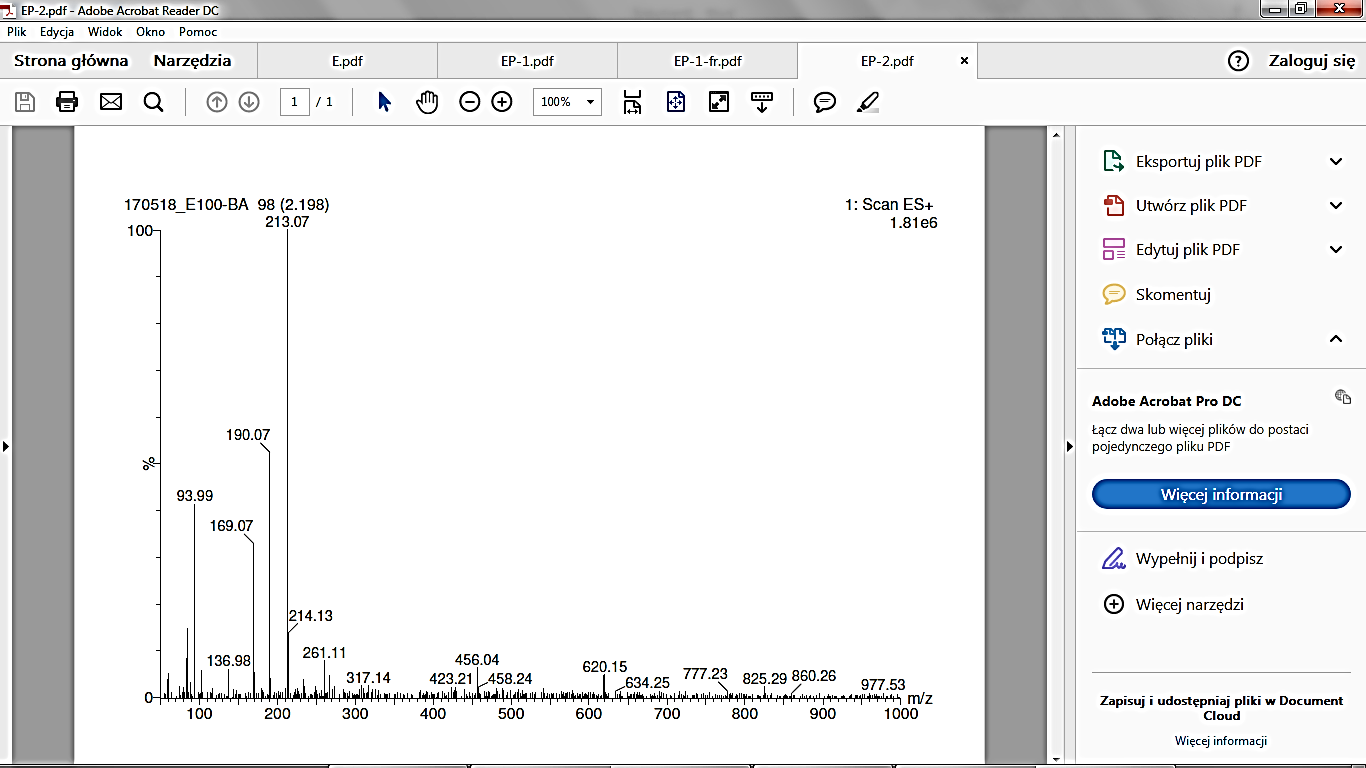


Fig. 4. MS spectrum of EP-2


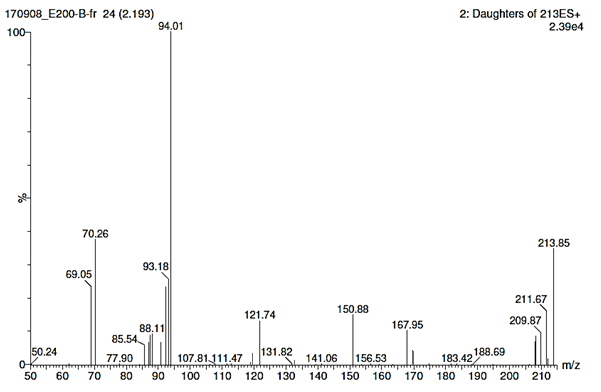


Fig. 5. MS/MS spectrum of EP-2


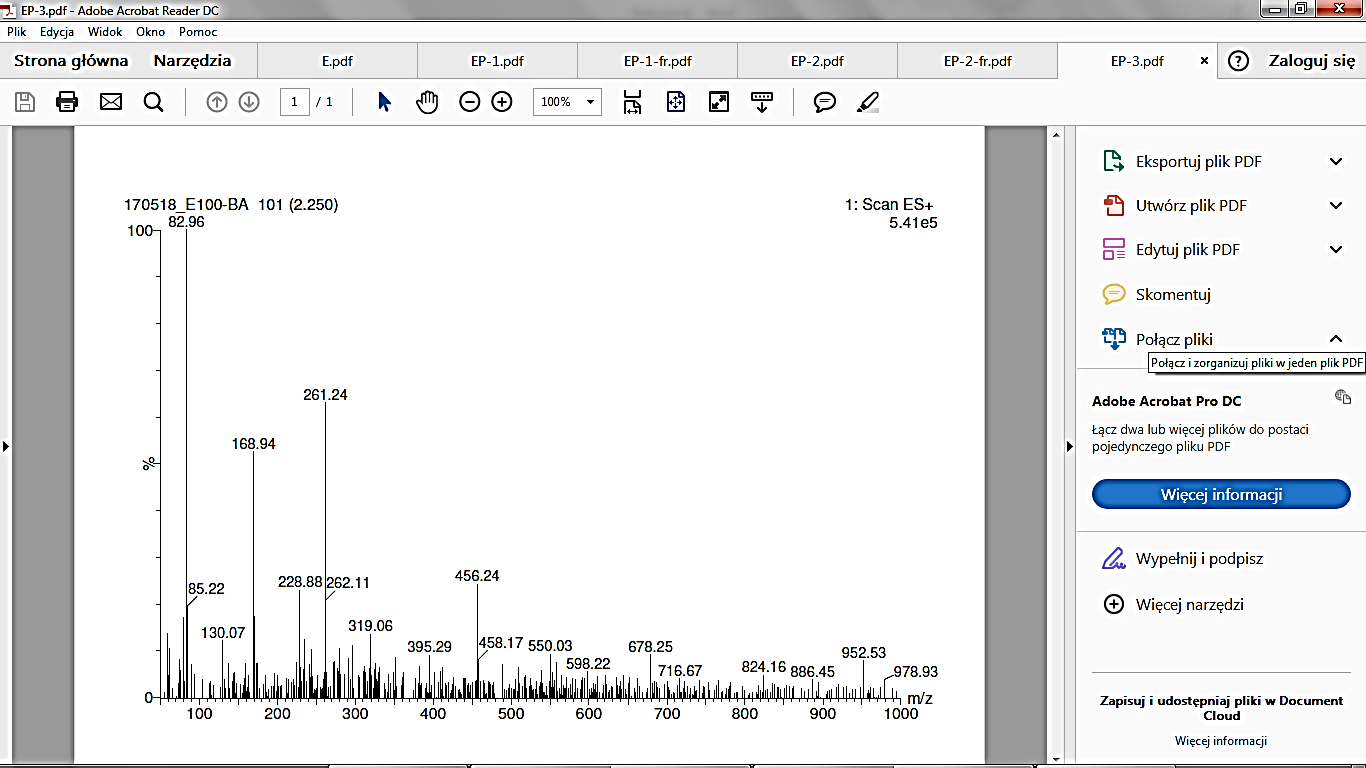


Fig. 6. MS spectrum of EP-3


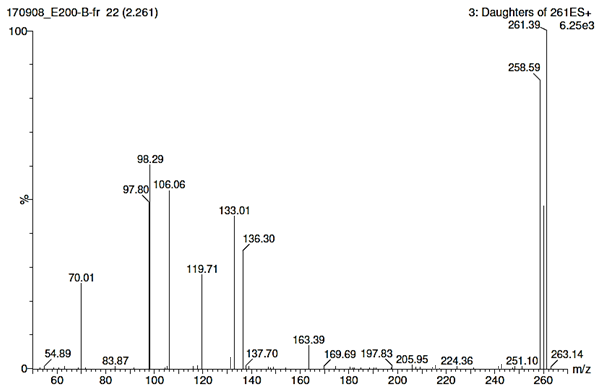


Fig. 7. MS/MS spectrum of EP-3


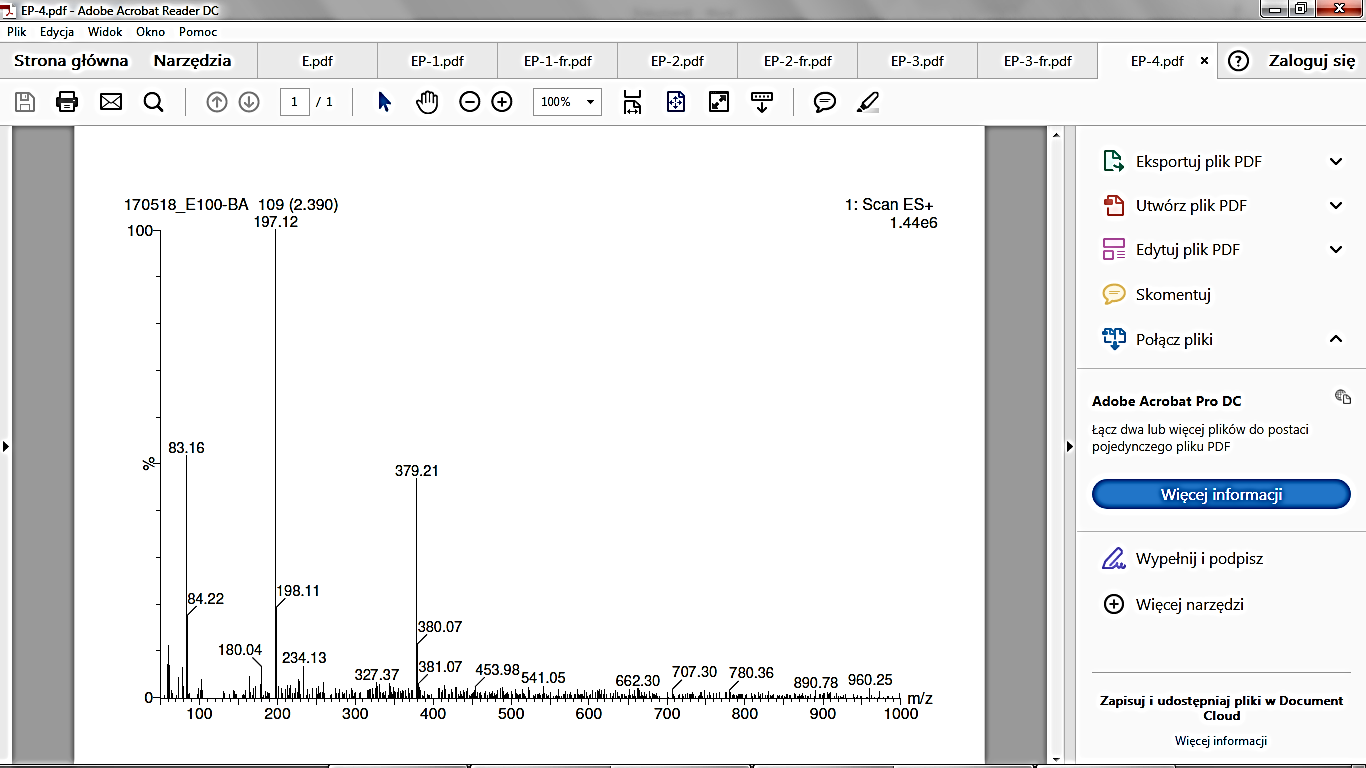


Fig. 8. MS spectrum of EP-4


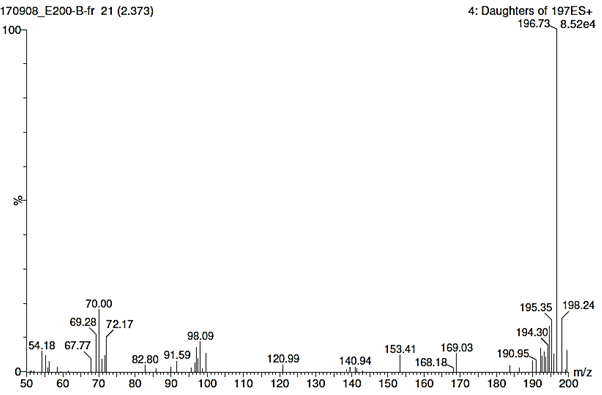


Fig. 9. MS/MS spectrum of EP-4


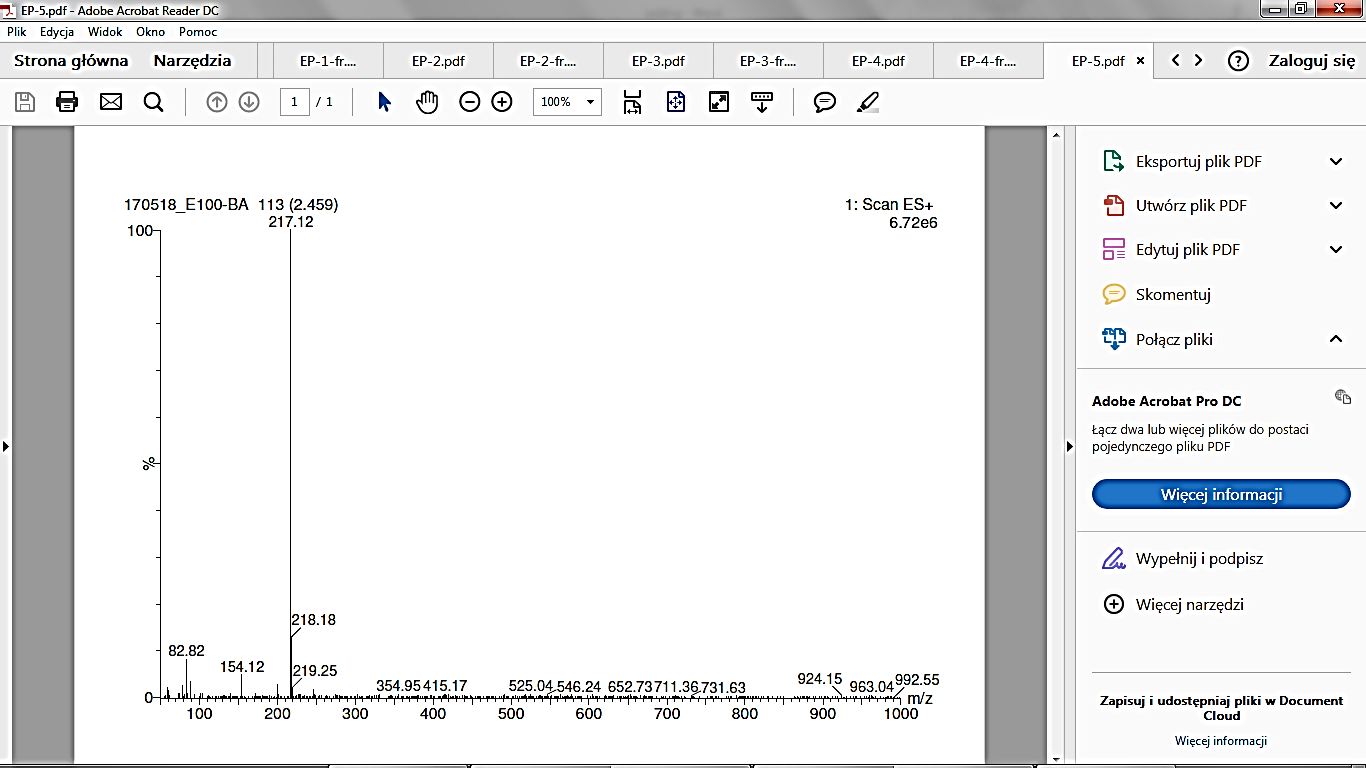


Fig. 10. MS spectrum of EP-5


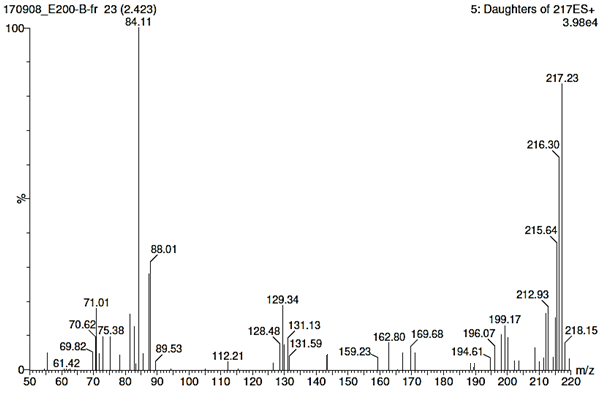


Fig. 11. MS/MS spectrum of EP-5


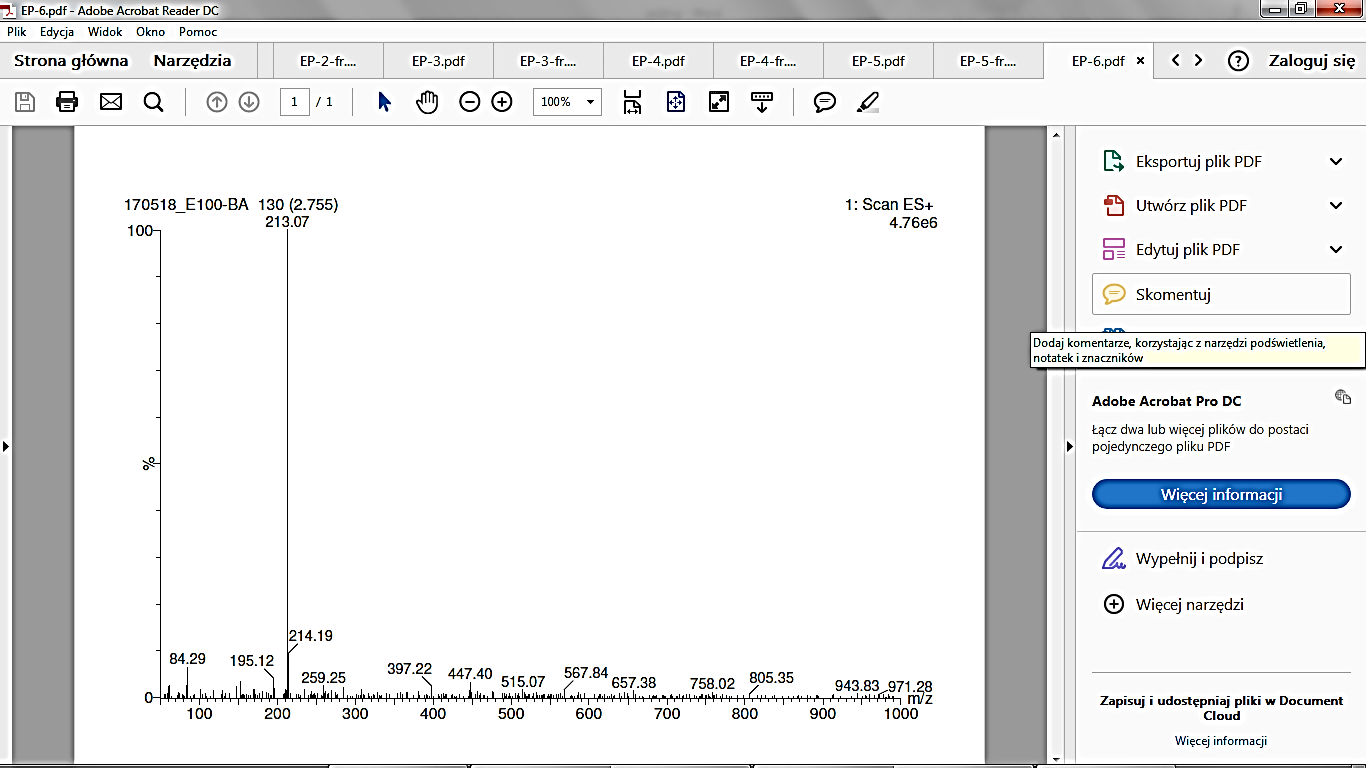


Fig. 12. MS spectrum of EP-6


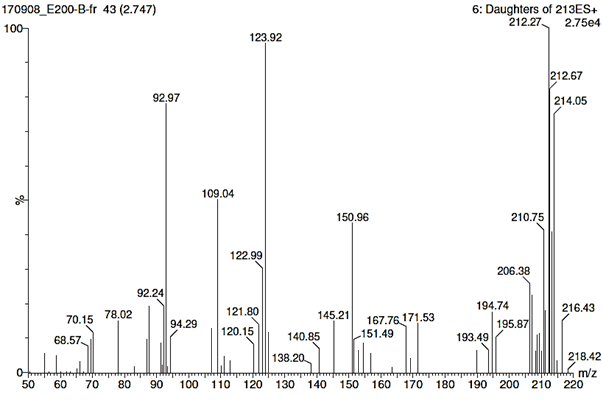


Fig. 13. MS/MS spectrum of EP-6


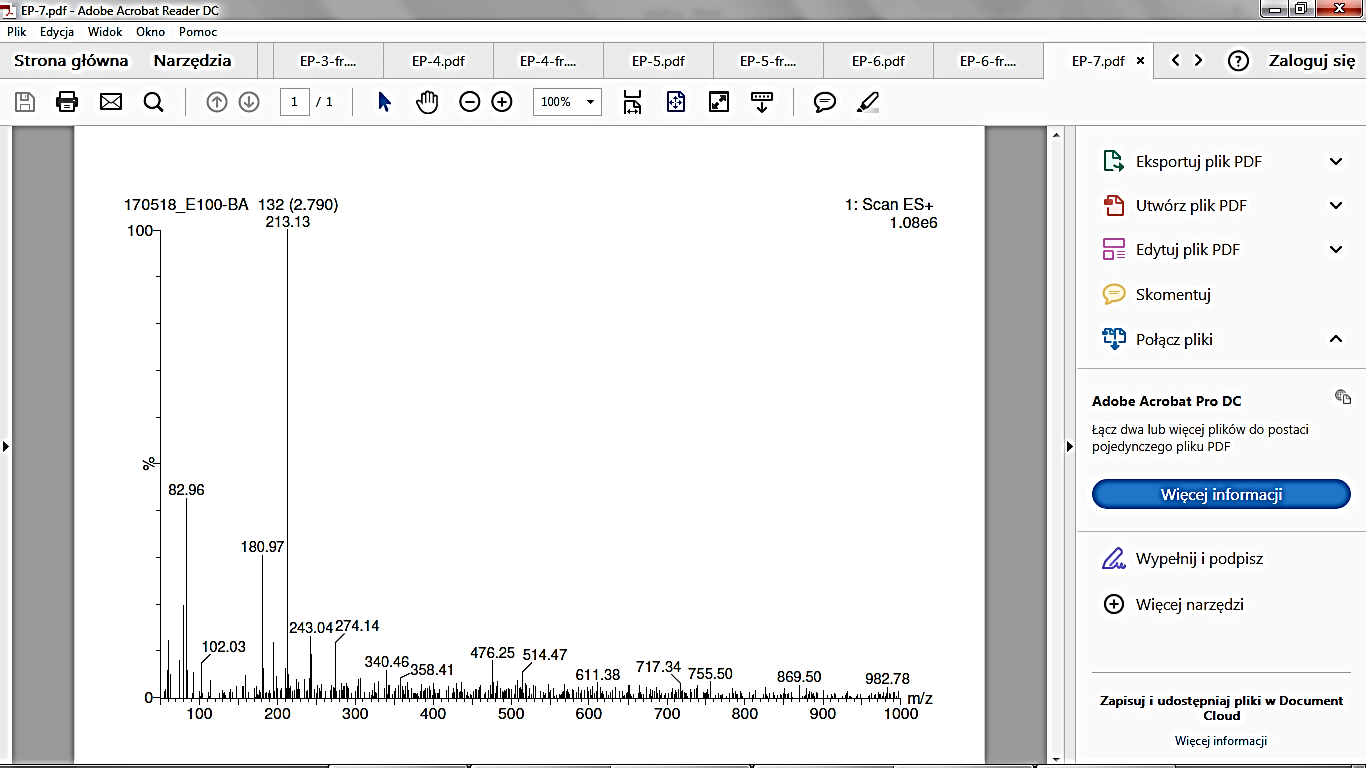


Fig. 14. MS spectrum of EP-7


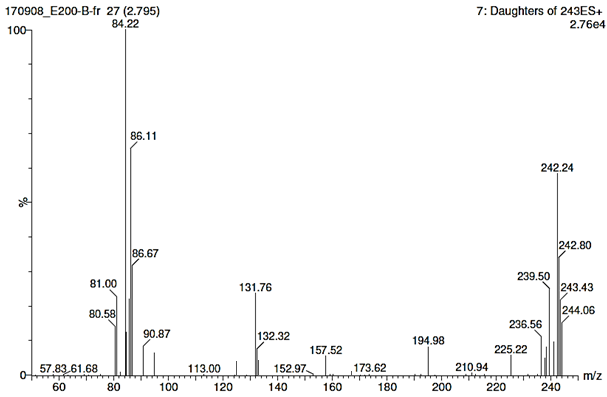


Fig. 15. MS/MS spectrum of EP-7


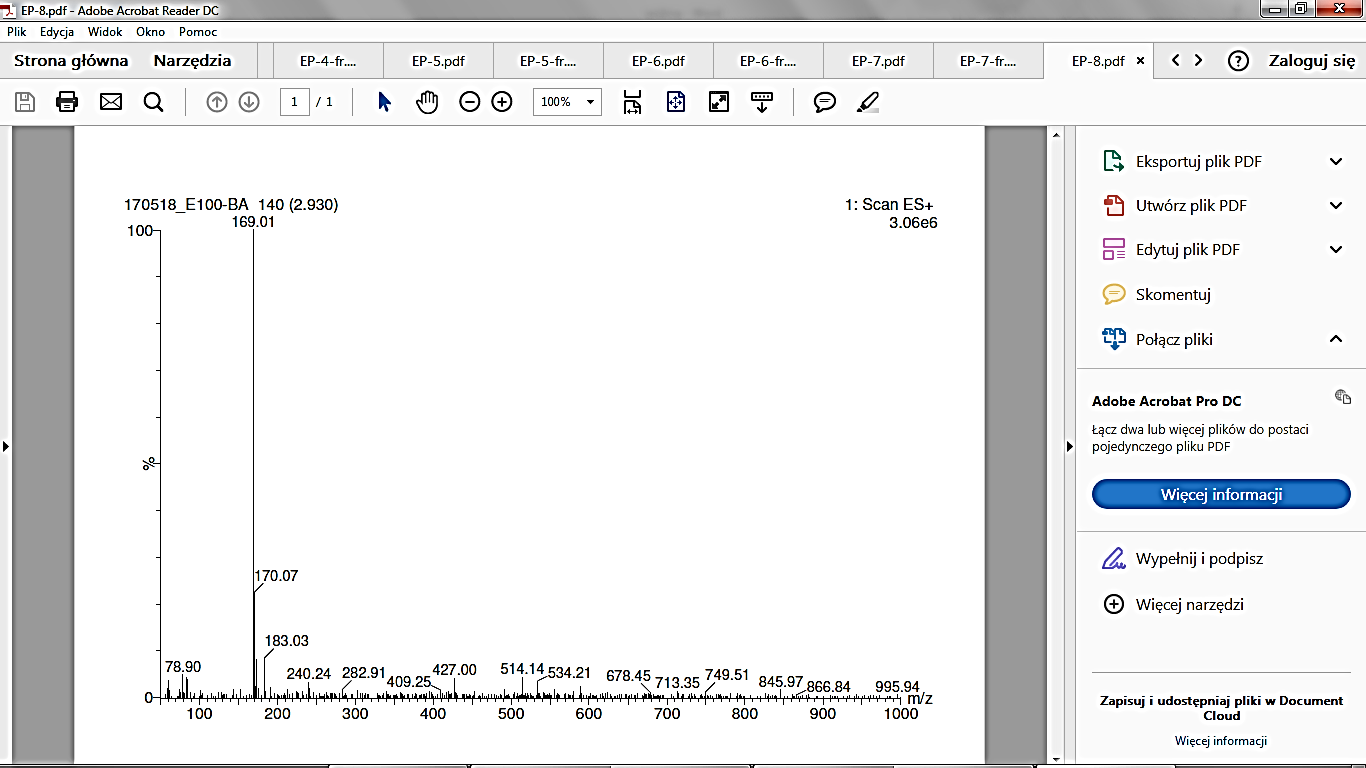


Fig. 16. MS spectrum of EP-8


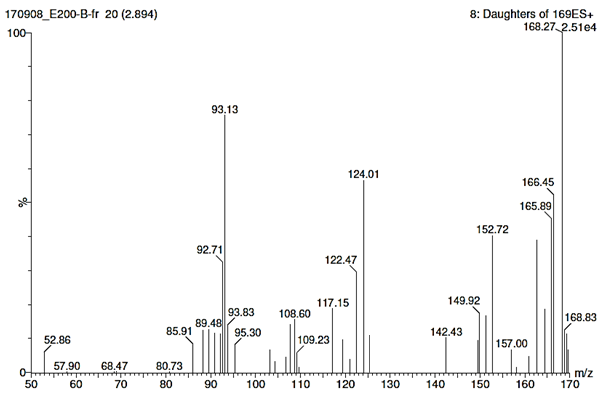


Fig. 17. MS/MS spectrum of EP-8


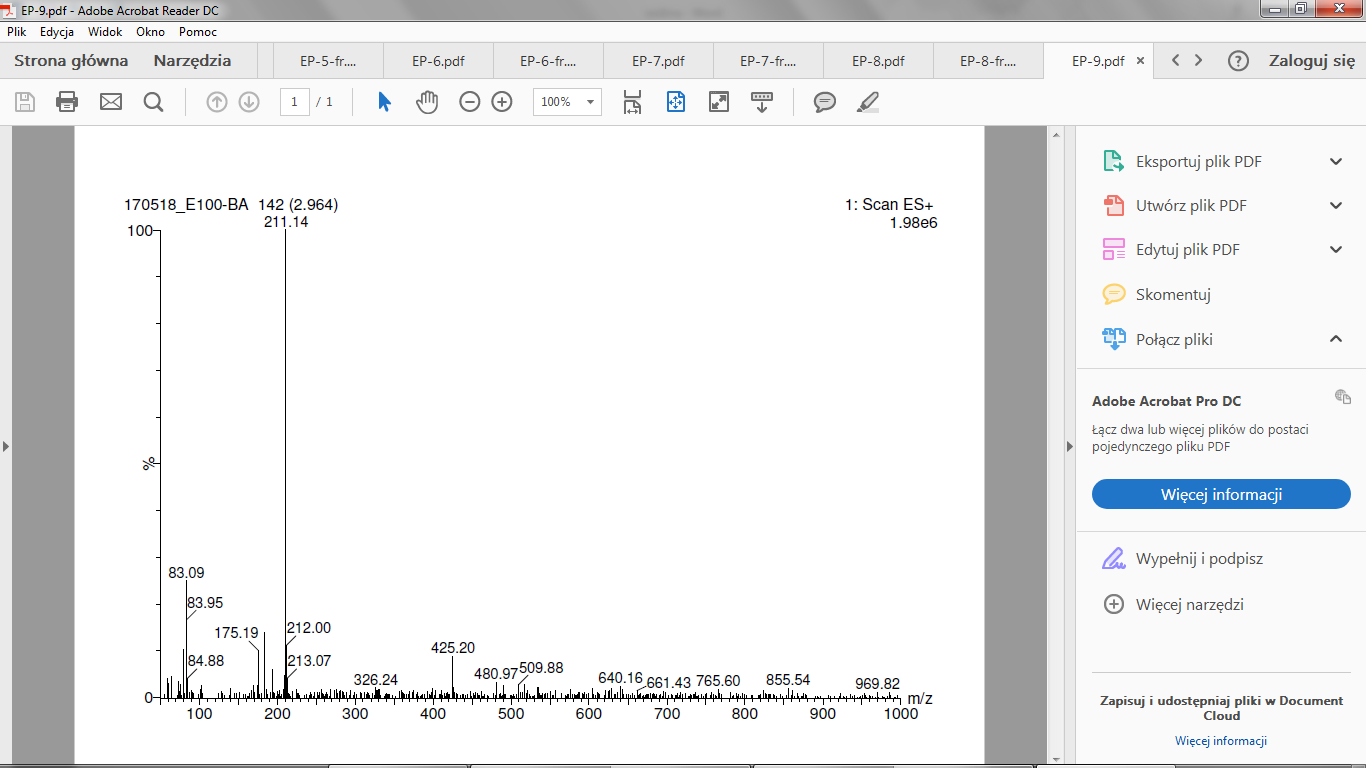


Fig. 18. MS spectrum of EP-9


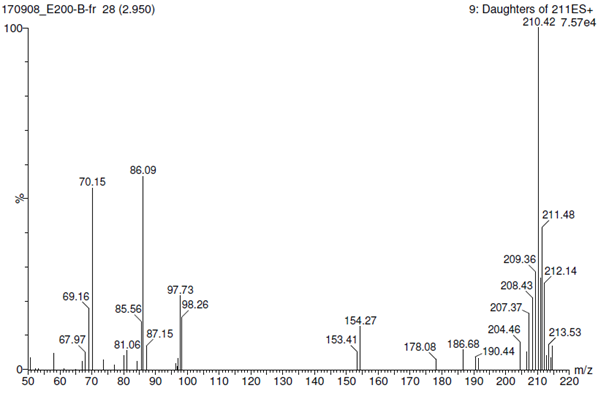


Fig. 19. MS/MS spectrum of EP-9


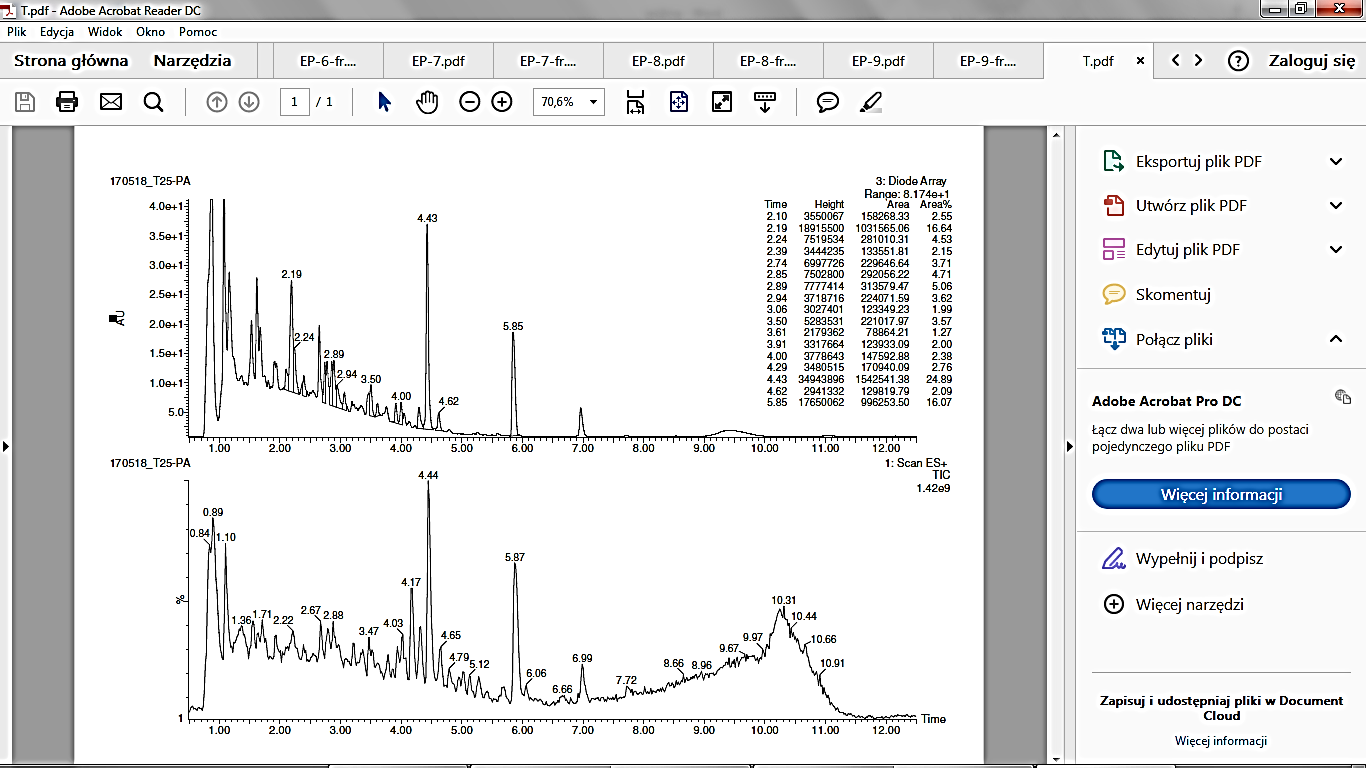


Fig. 20. Chromatogram of mycelium extract after addition of testosterone


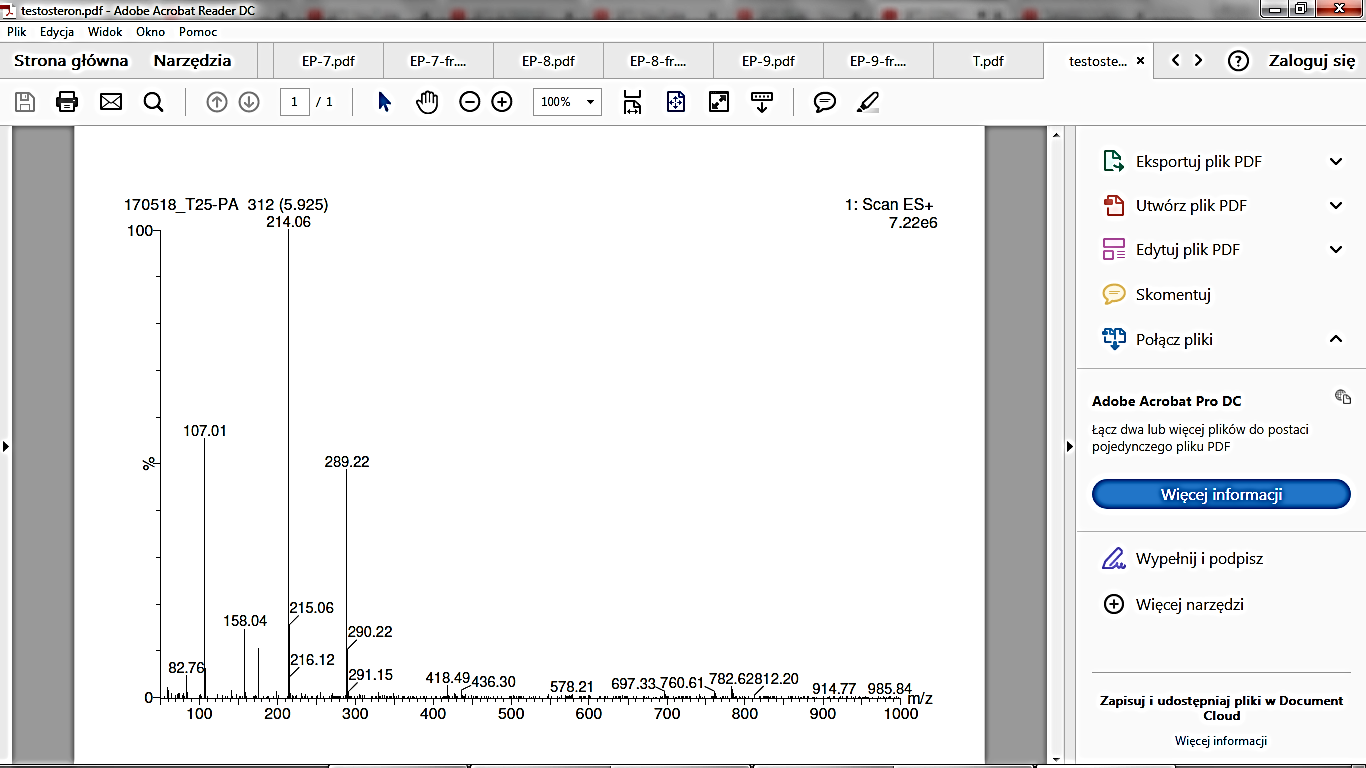


Fig. 21. MS spectrum of testosterone


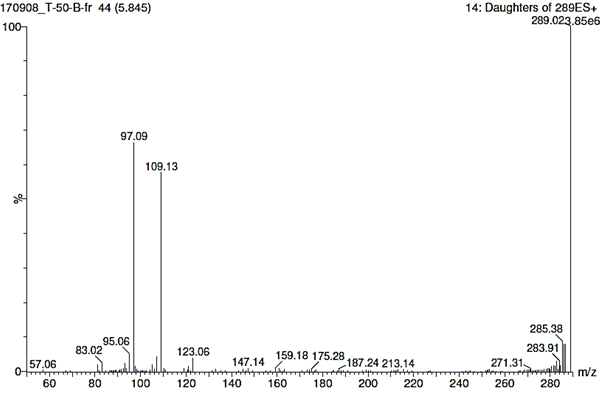


Fig. 22. MS/MS spectrum of testosterone


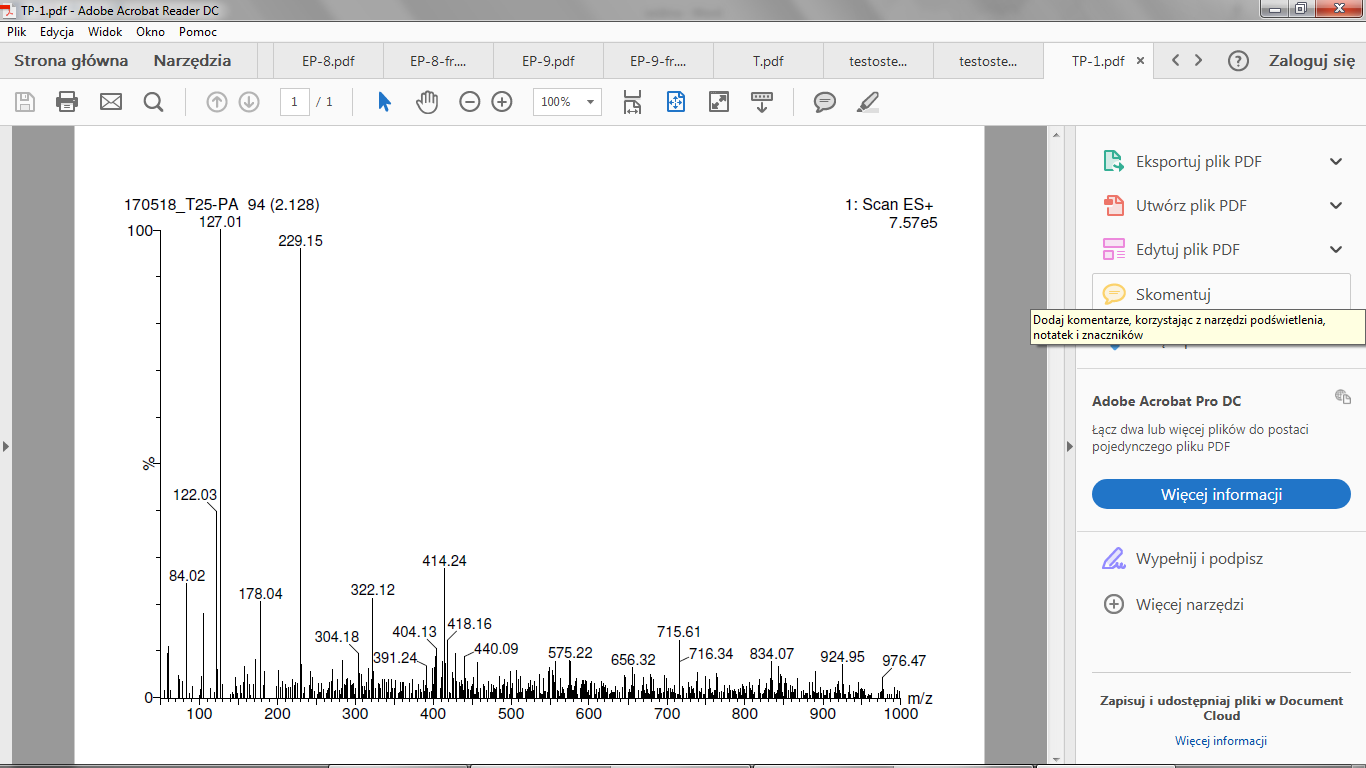


Fig. 23. MS spectrum of TP-1


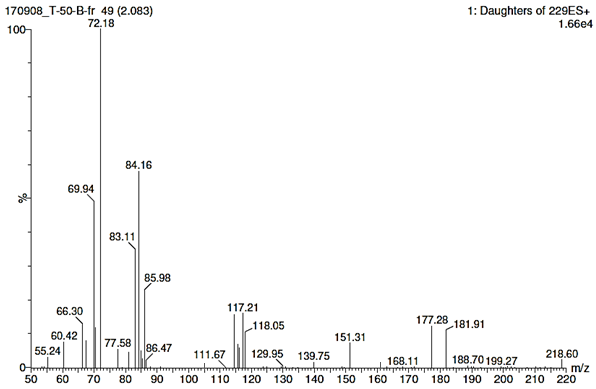


Fig. 24. MS/MS spectrum of TP-1


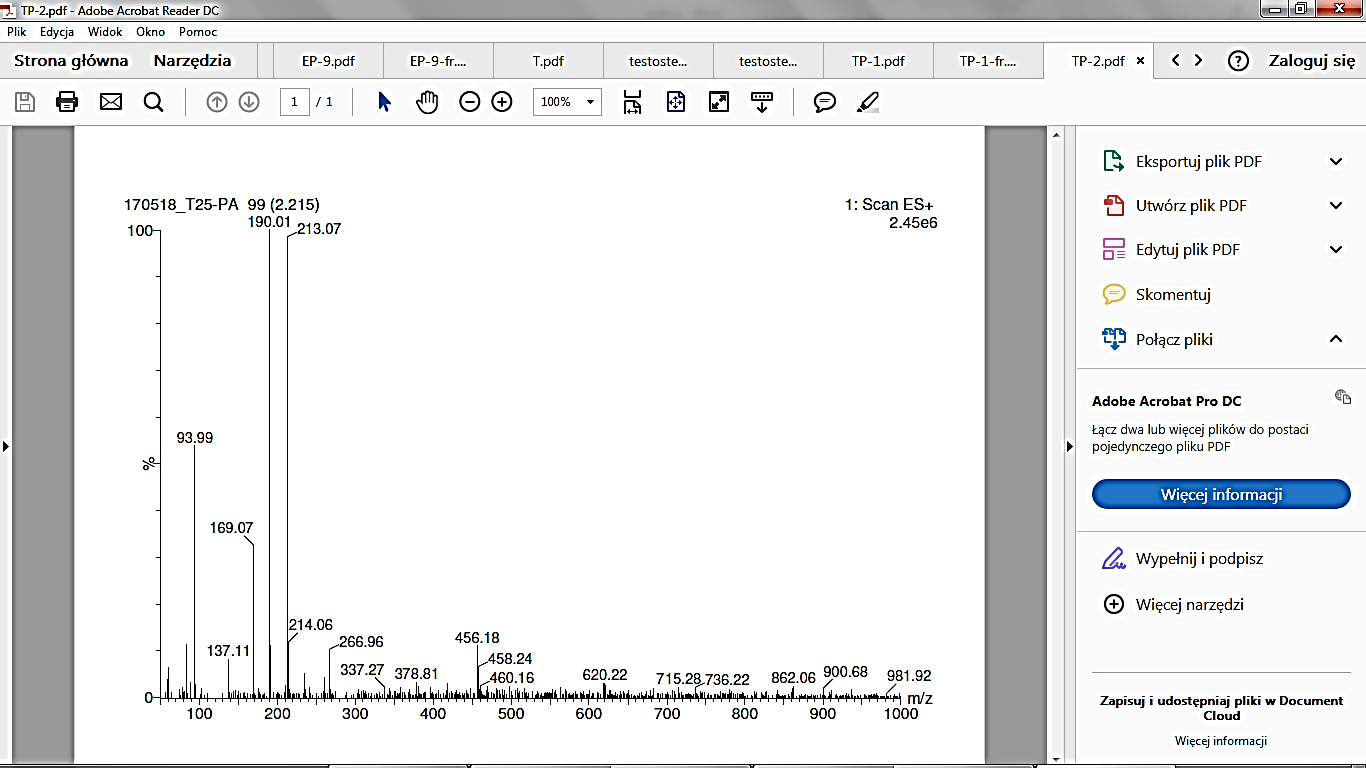


Fig. 25. MS spectrum of TP-2


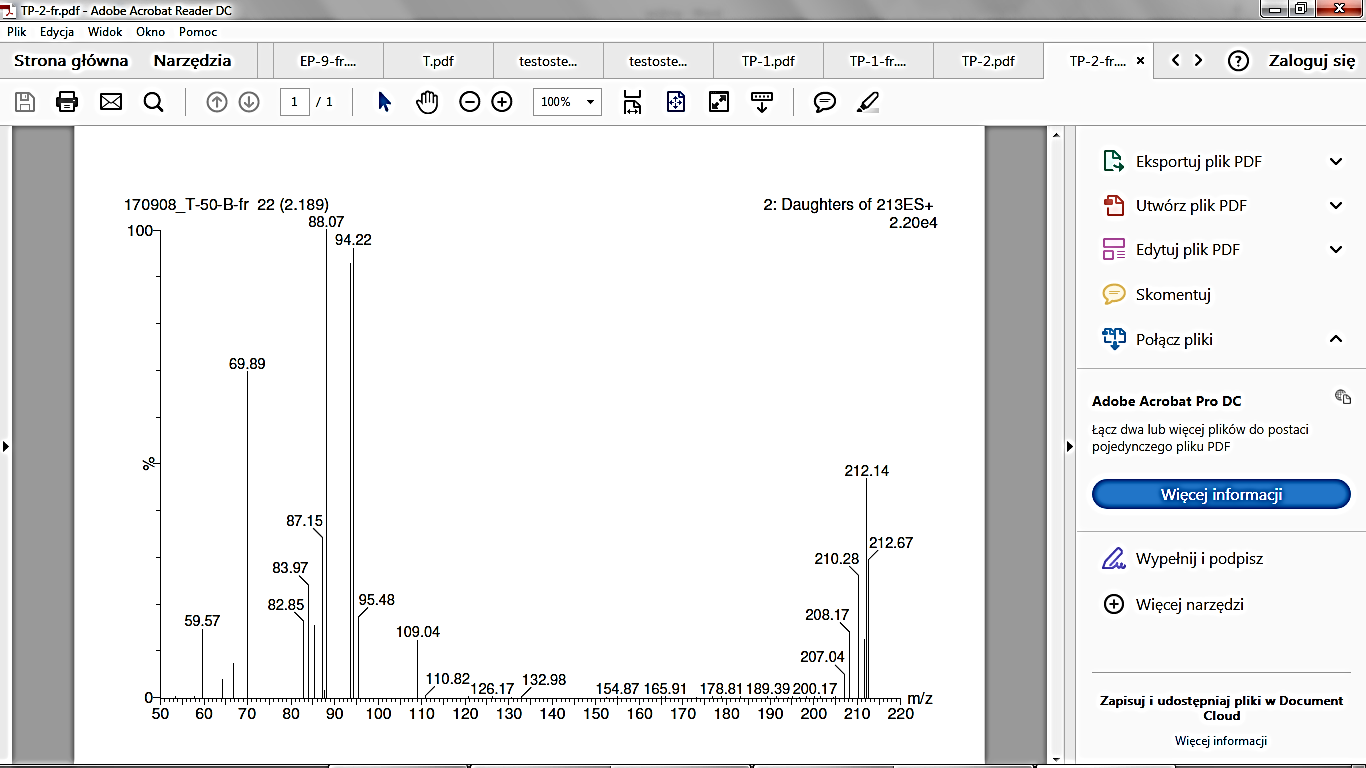


Fig. 26. MS/MS spectrum of TP-2


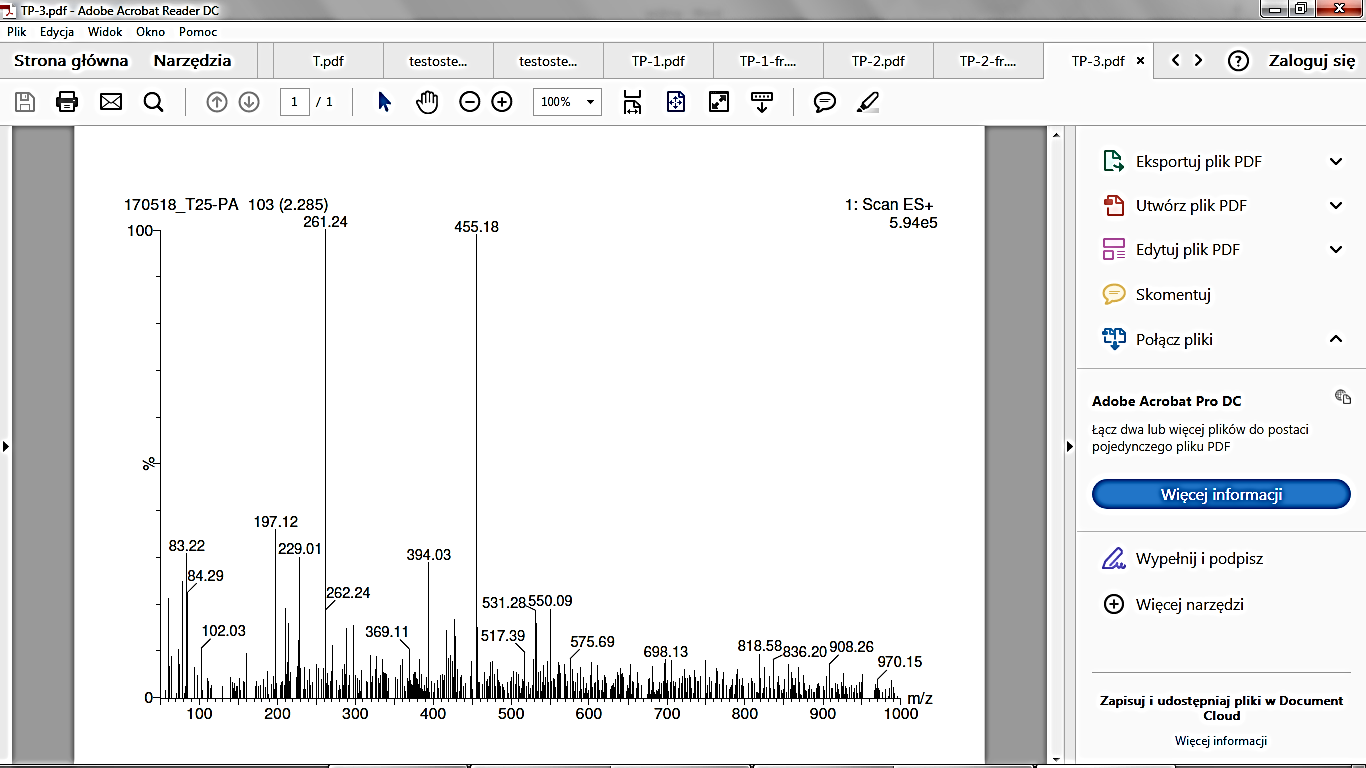


Fig. 27. MS spectrum of TP-3


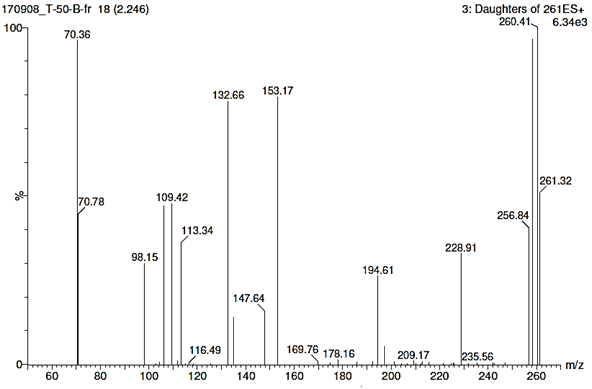


Fig. 28. MS/MS spectrum of TP-3


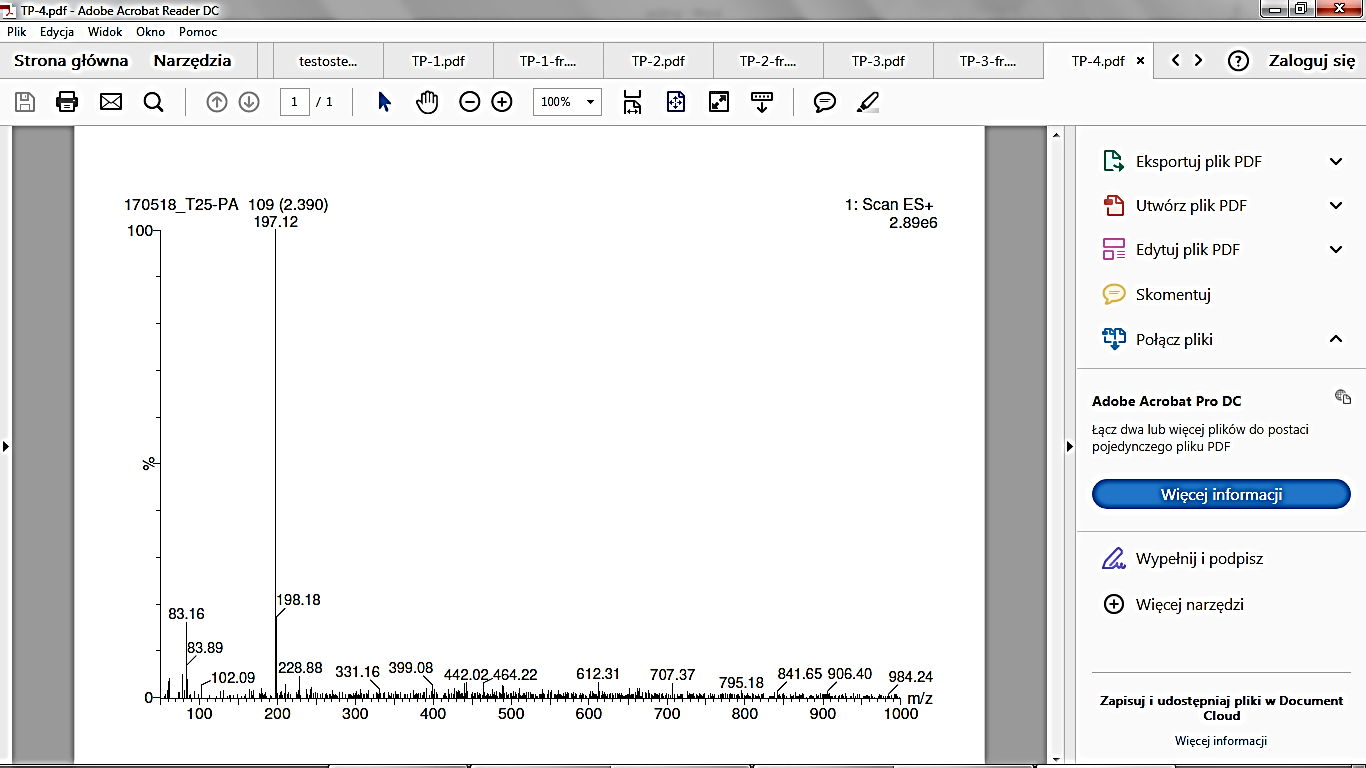


Fig. 29. MS spectrum of TP-4


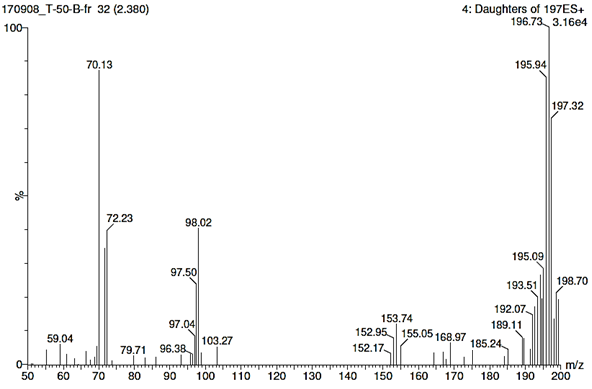


Fig. 30. MS/MS spectrum of TP-4


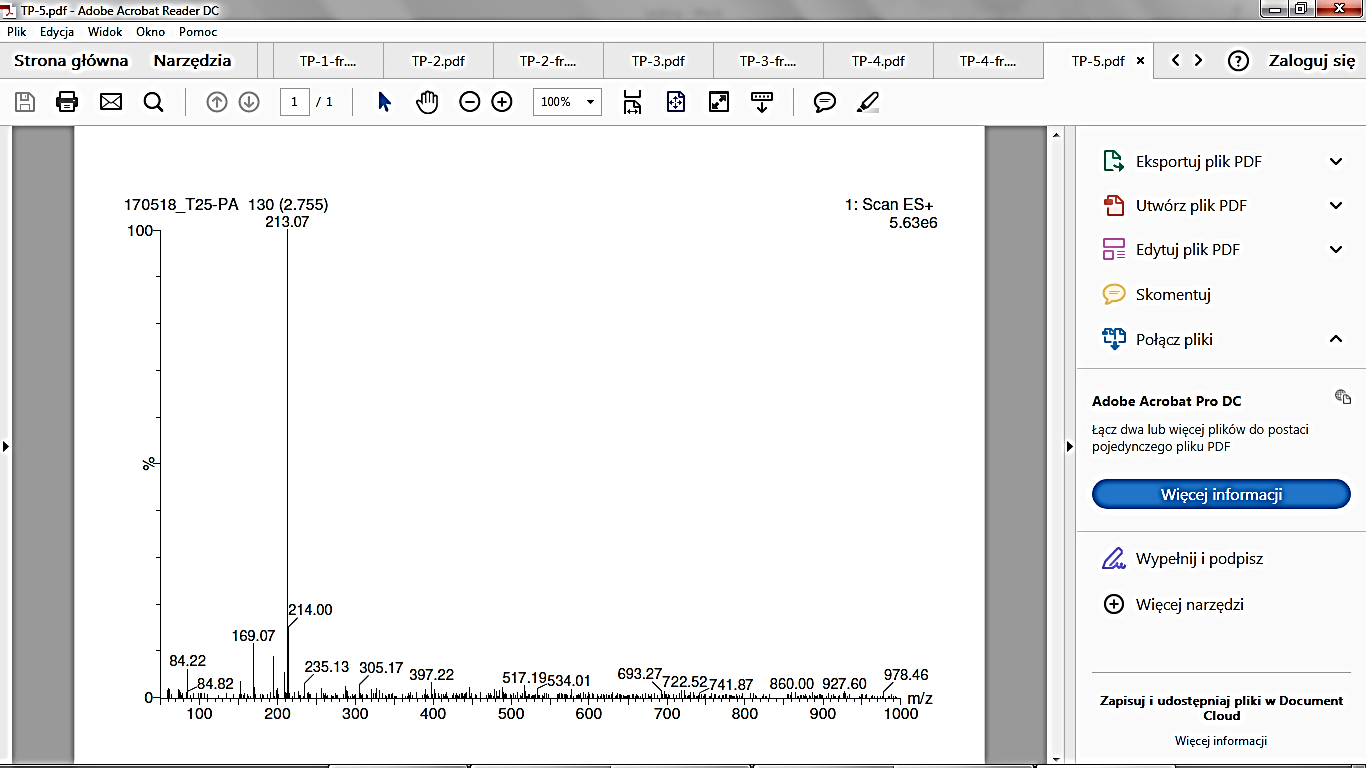


Fig. 31. MS spectrum of TP-5


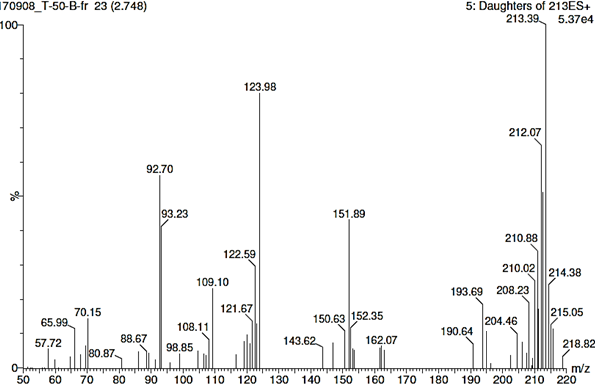


Fig. 32. MS/MS spectrum of TP-5


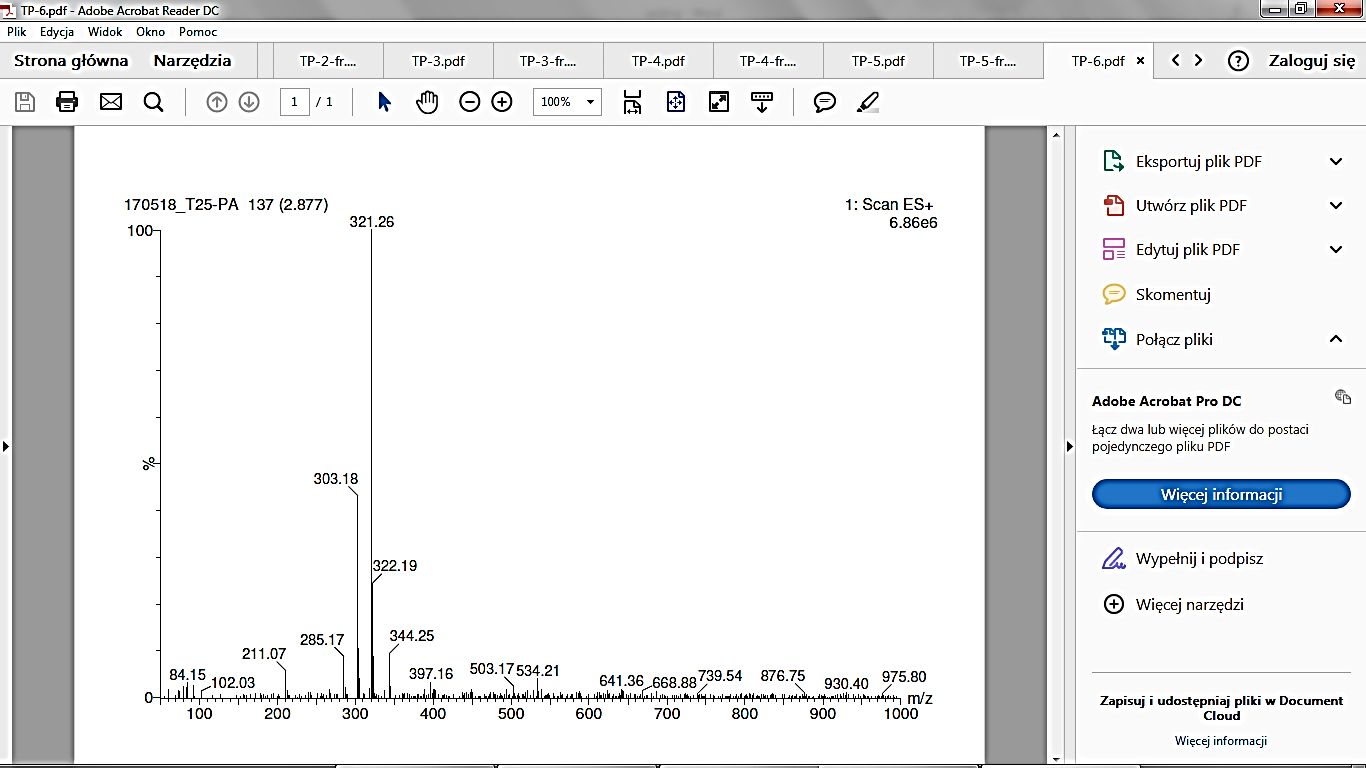


Fig. 33. MS spectrum of TP-6


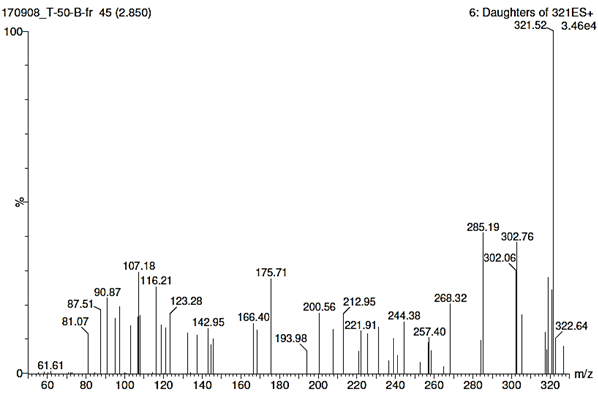


Fig. 34. MS/MS spectrum of TP-6


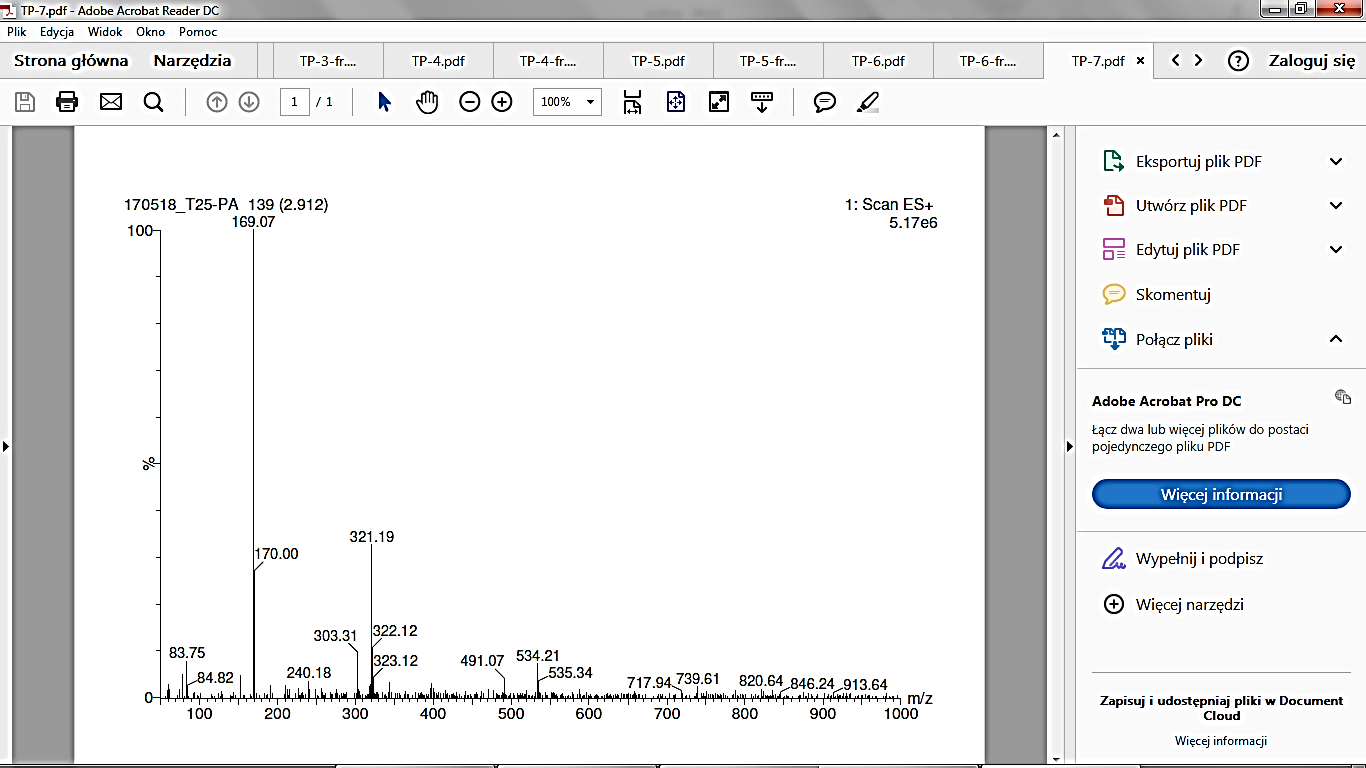


Fig. 35. MS spectrum of TP-7


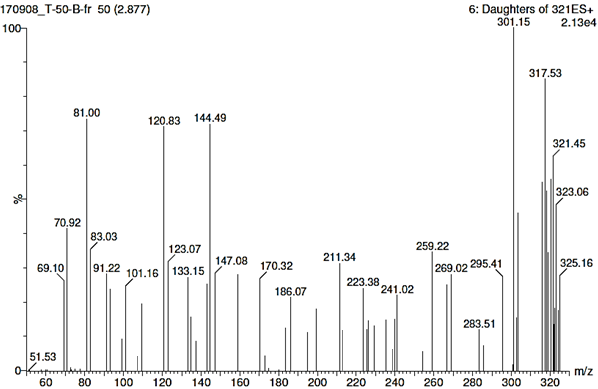


Fig. 36. MS/MS spectrum of TP-7


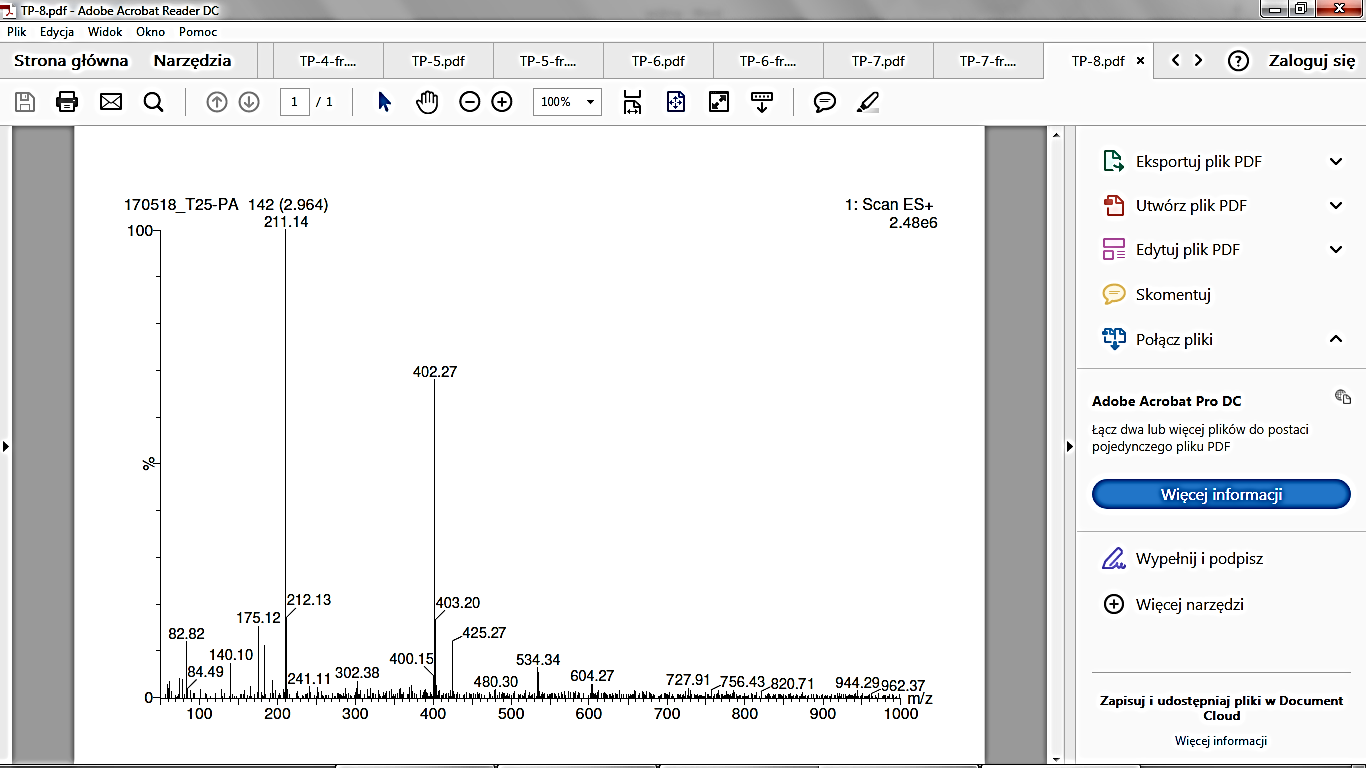


Fig. 37. MS spectrum of TP-8


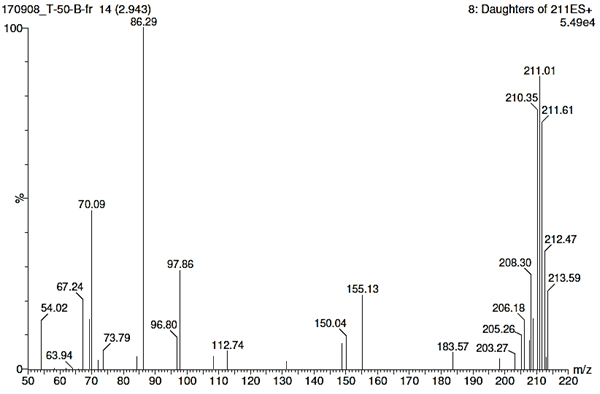


Fig. 38. MS/MS spectrum of TP-8


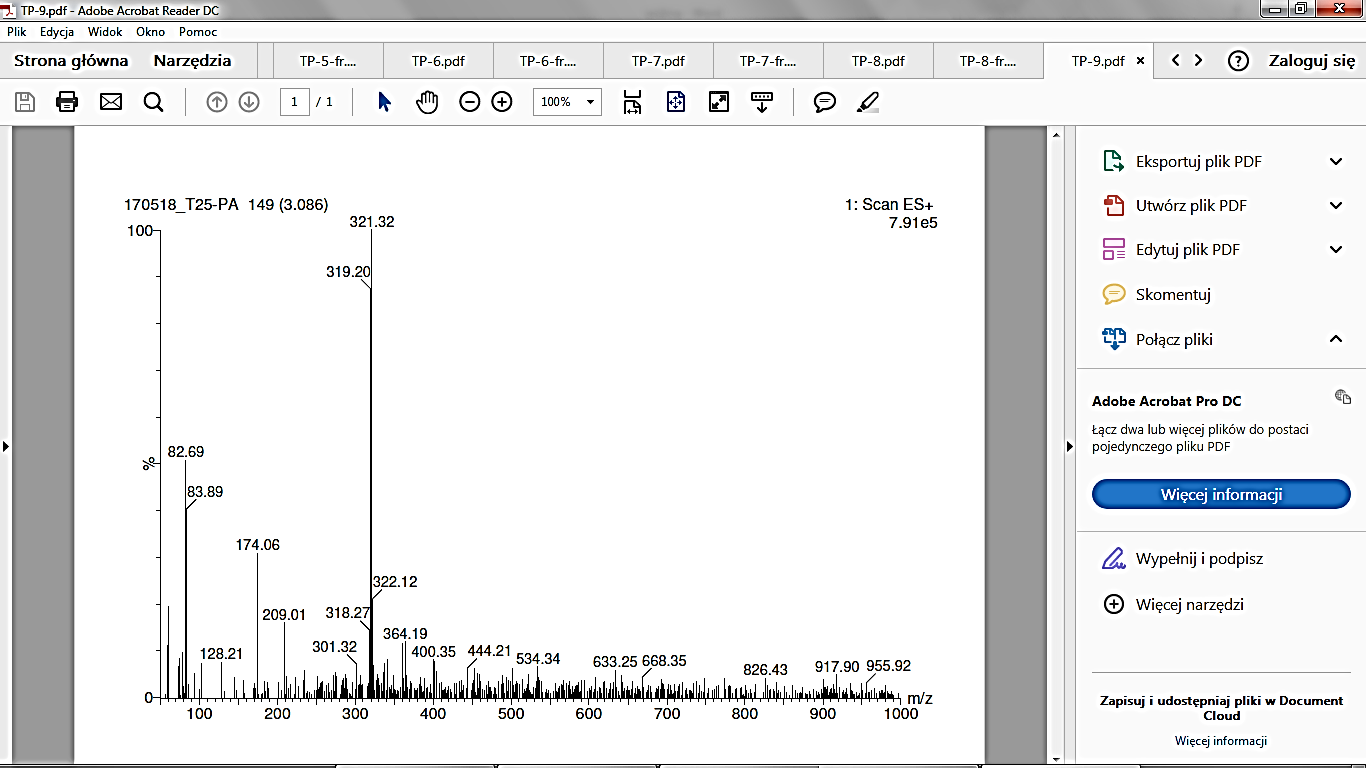


Fig. 39. MS spectrum of TP-9


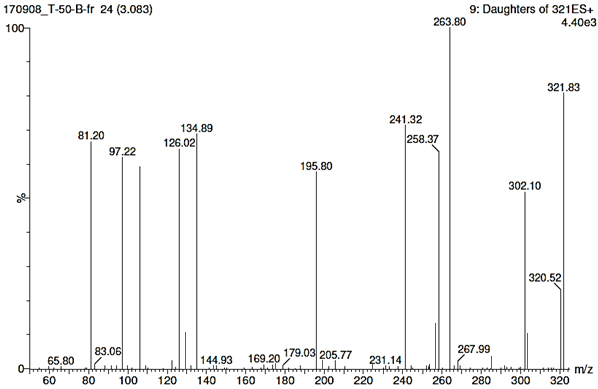


Fig. 40. MS/MS spectrum of TP-9


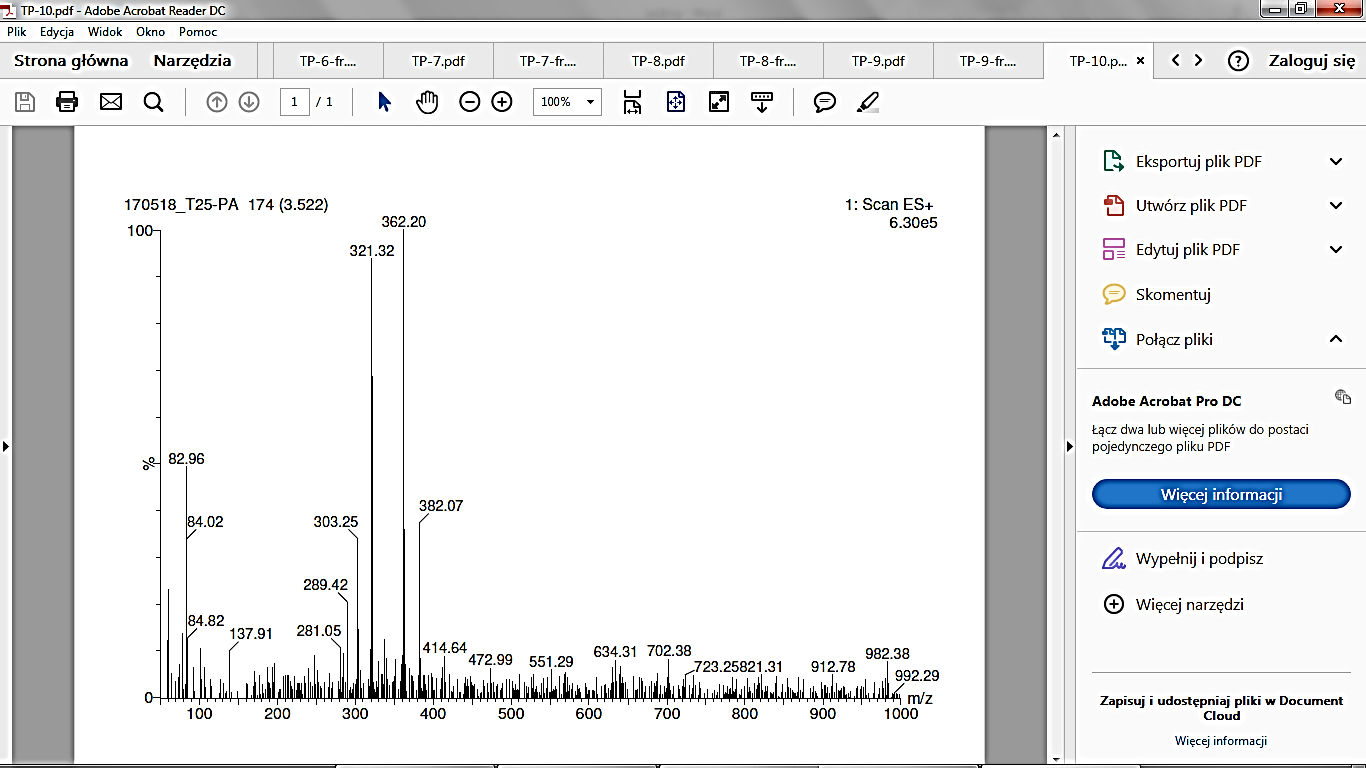


Fig. 41. MS spectrum of TP-10


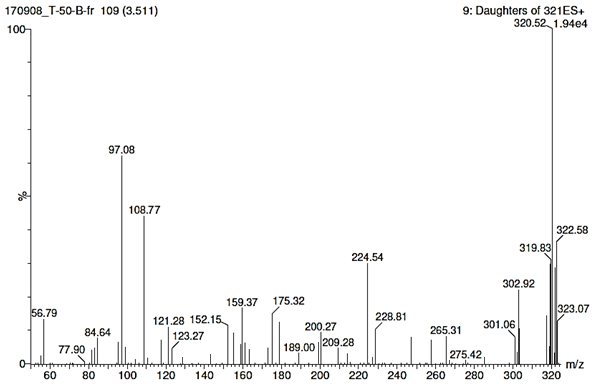


Fig. 42. MS/MS spectrum of TP-10


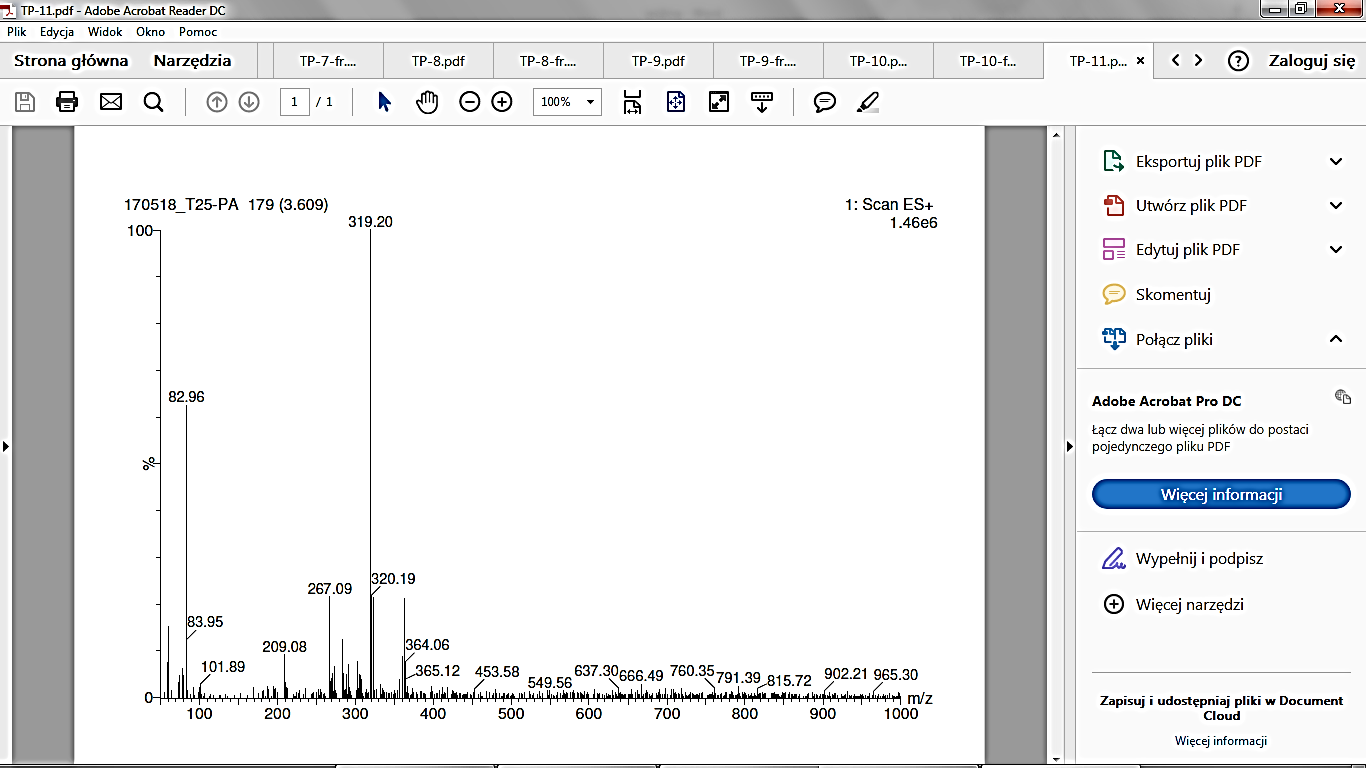


Fig. 43. MS spectrum of TP-11


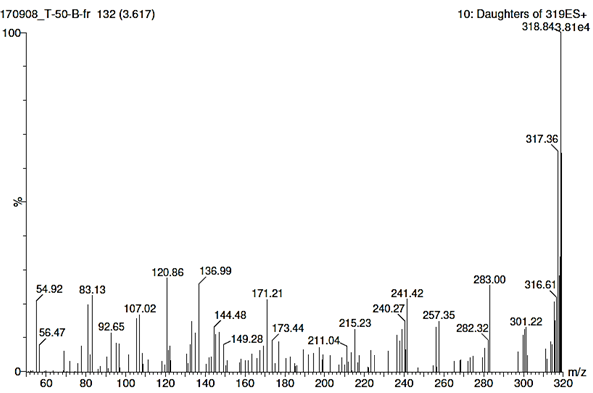


Fig. 44. MS/MS spectrum of TP-11


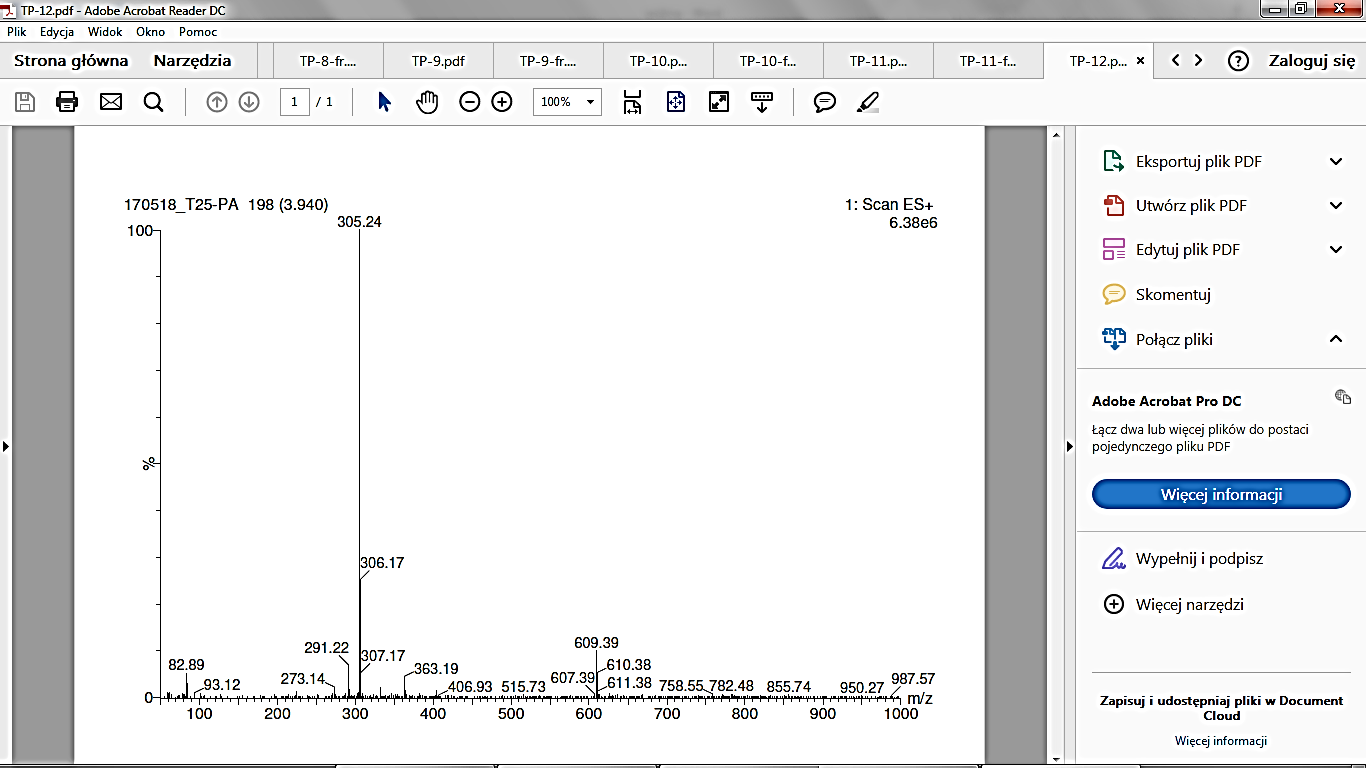


Fig. 45. MS spectrum of TP-12


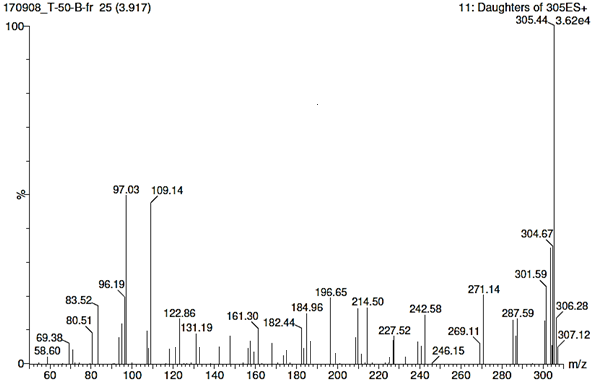


Fig. 46. MS/MS spectrum of TP-12


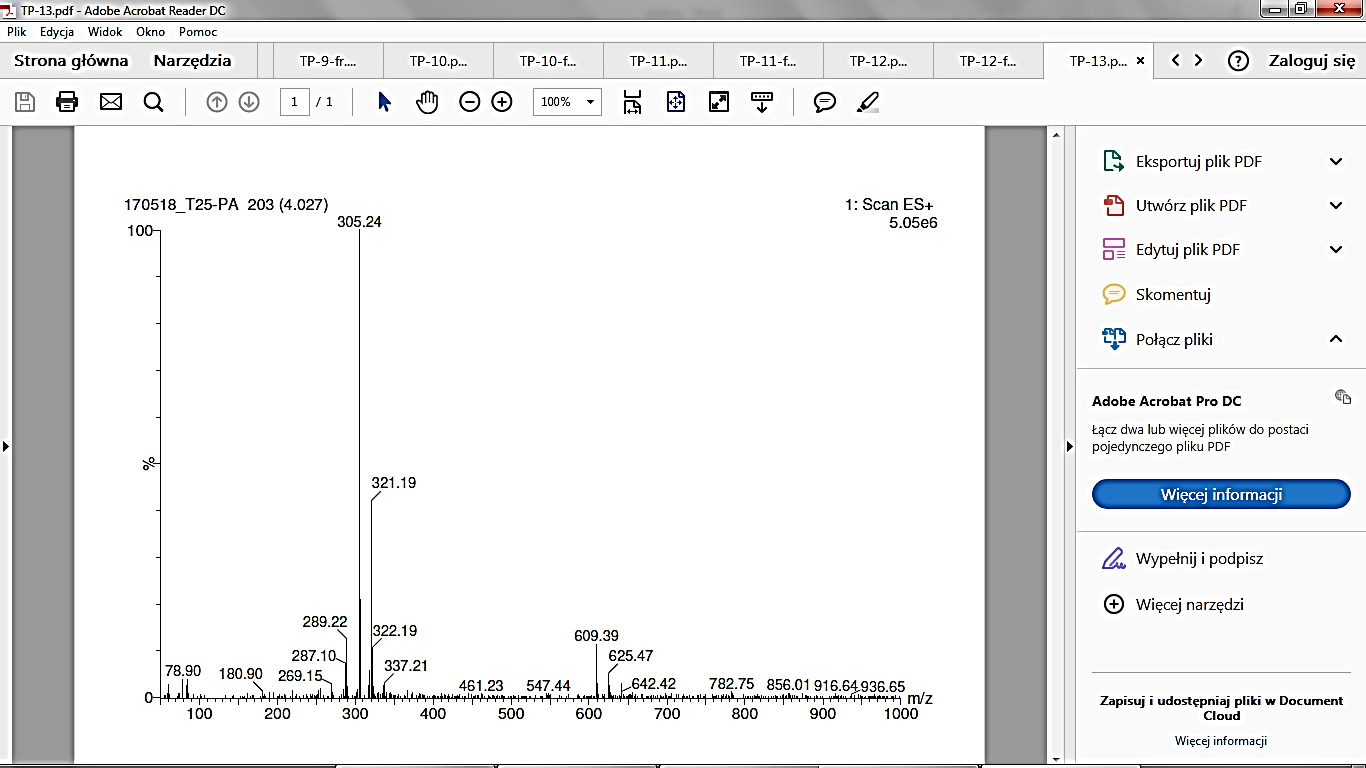


Fig. 47. MS spectrum of TP-13


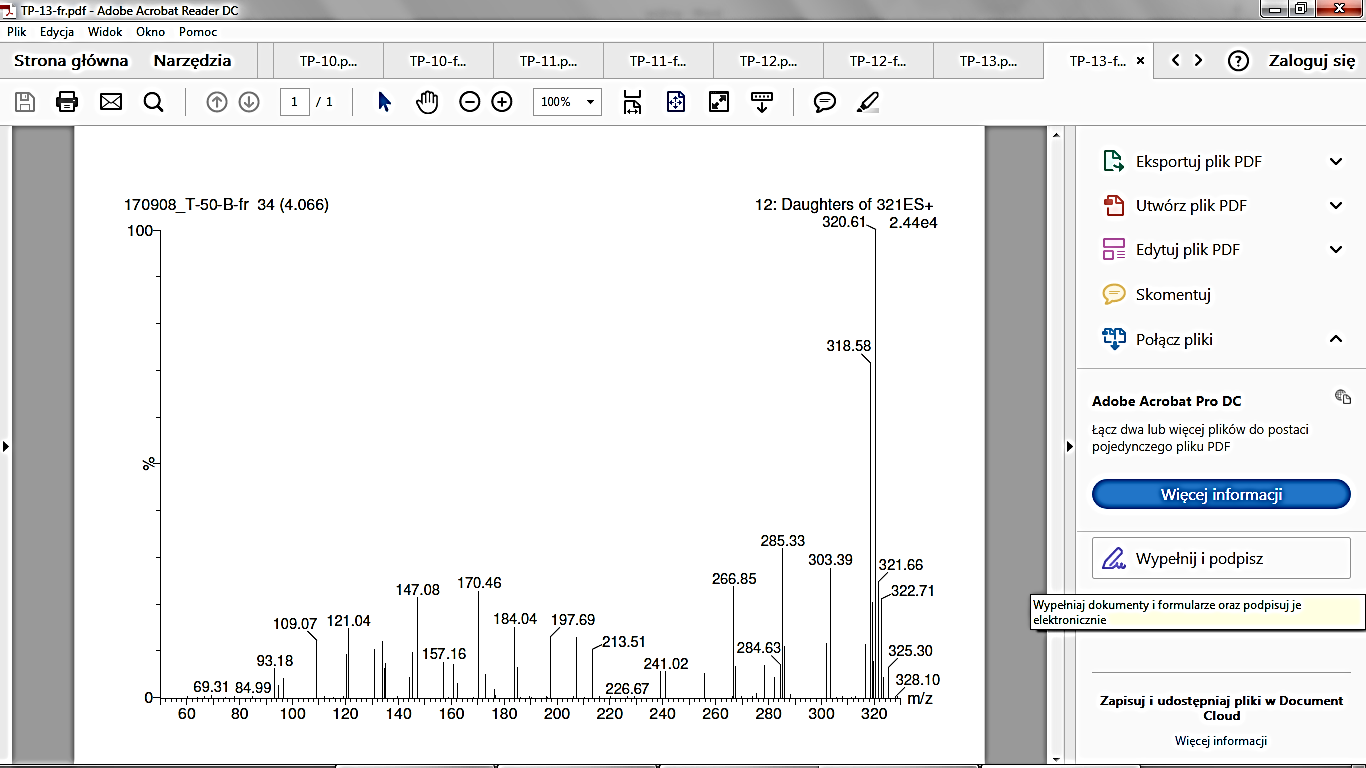


Fig. 48. MS/MS spectrum of TP-13


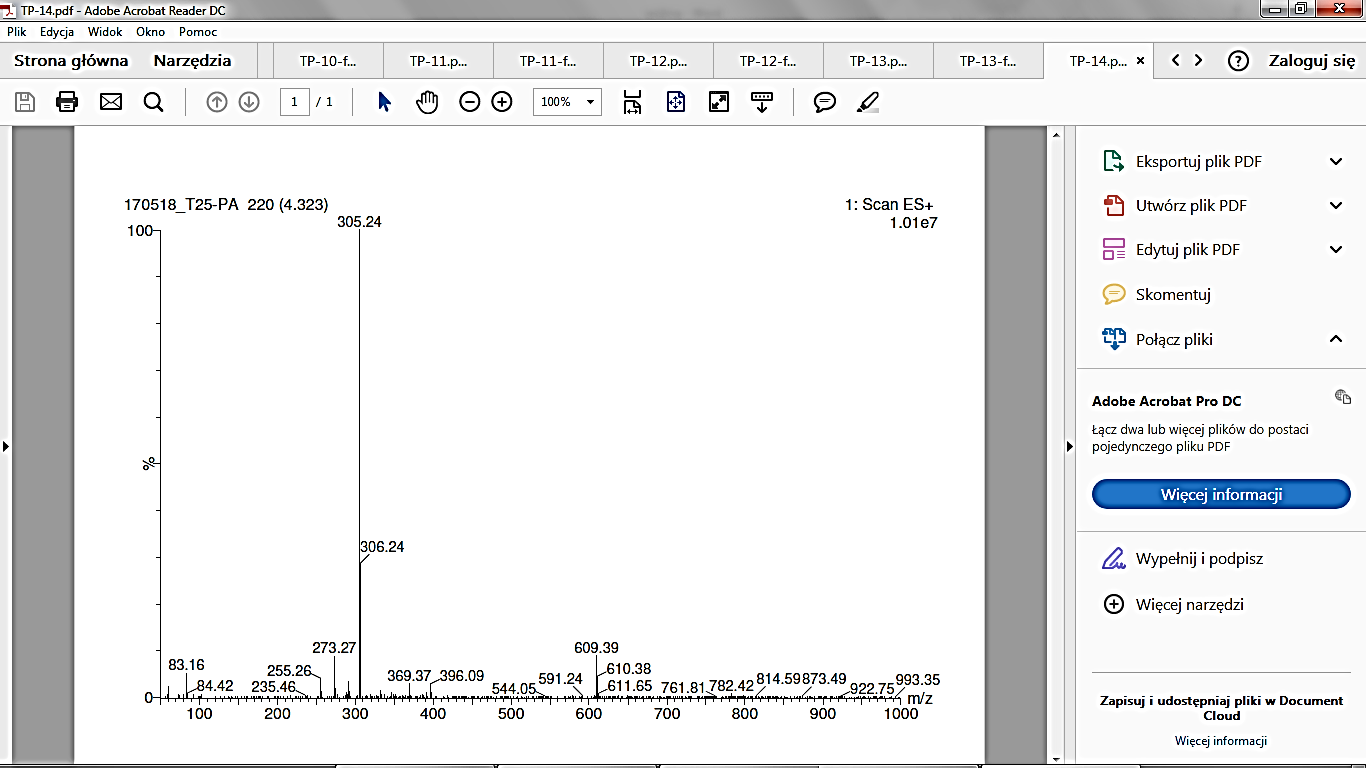


Fig. 49. MS spectrum of TP-14


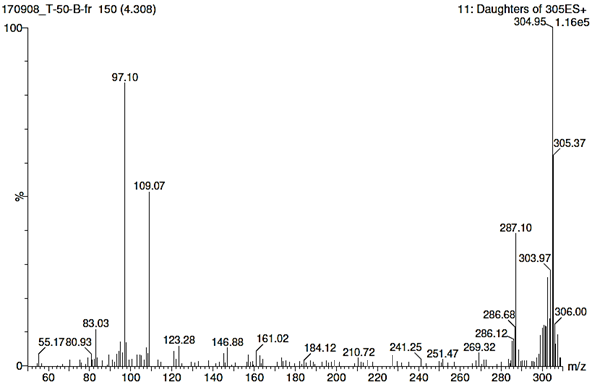


Fig. 50. MS/MS spectrum of TP-14


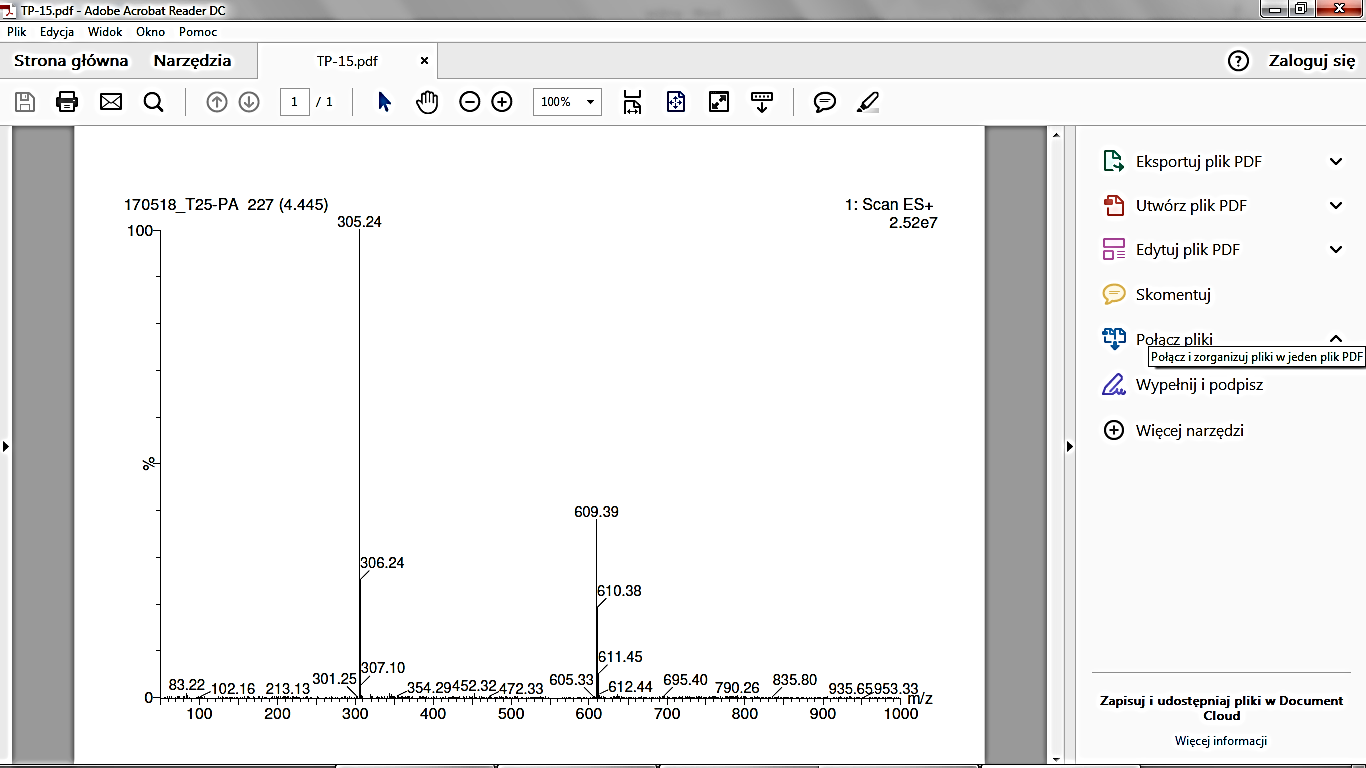


Fig. 51. MS spectrum of TP-15


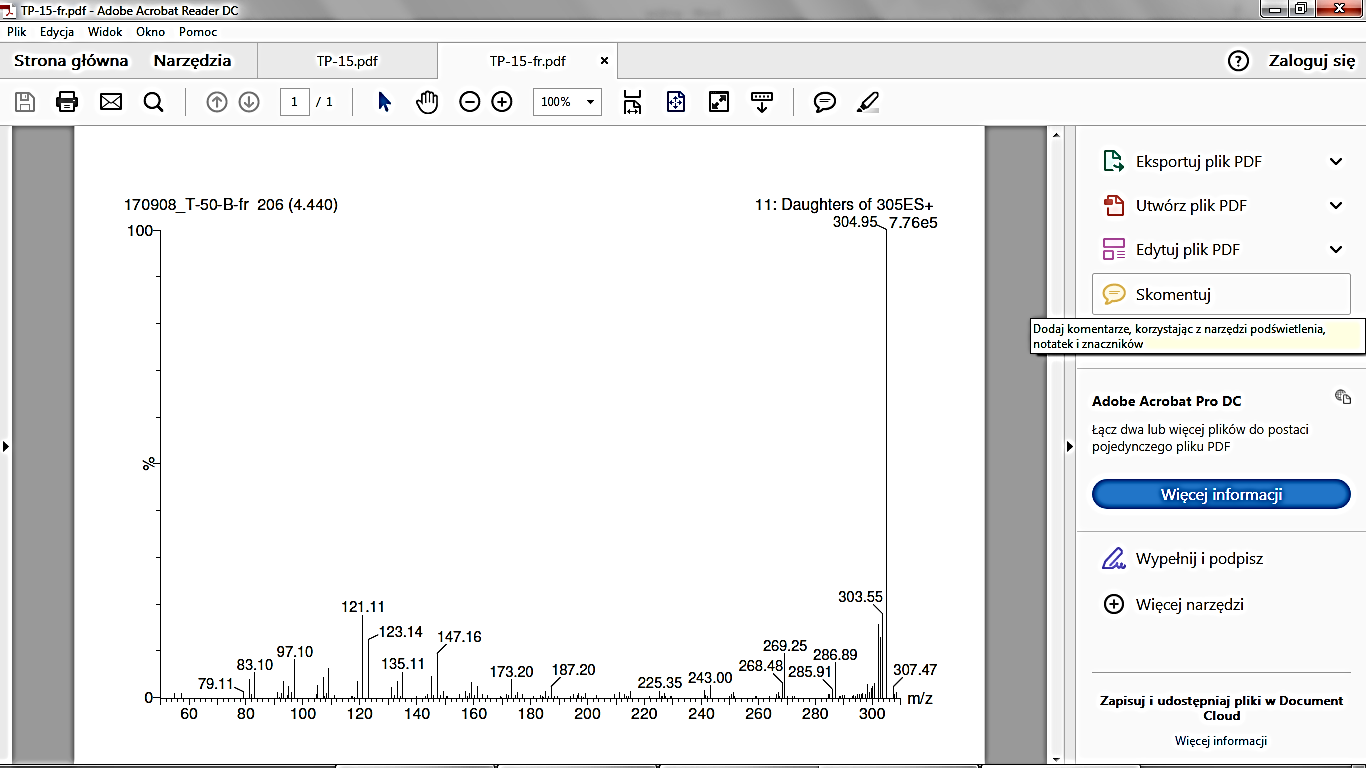


Fig. 52. MS/MS spectrum of TP-15


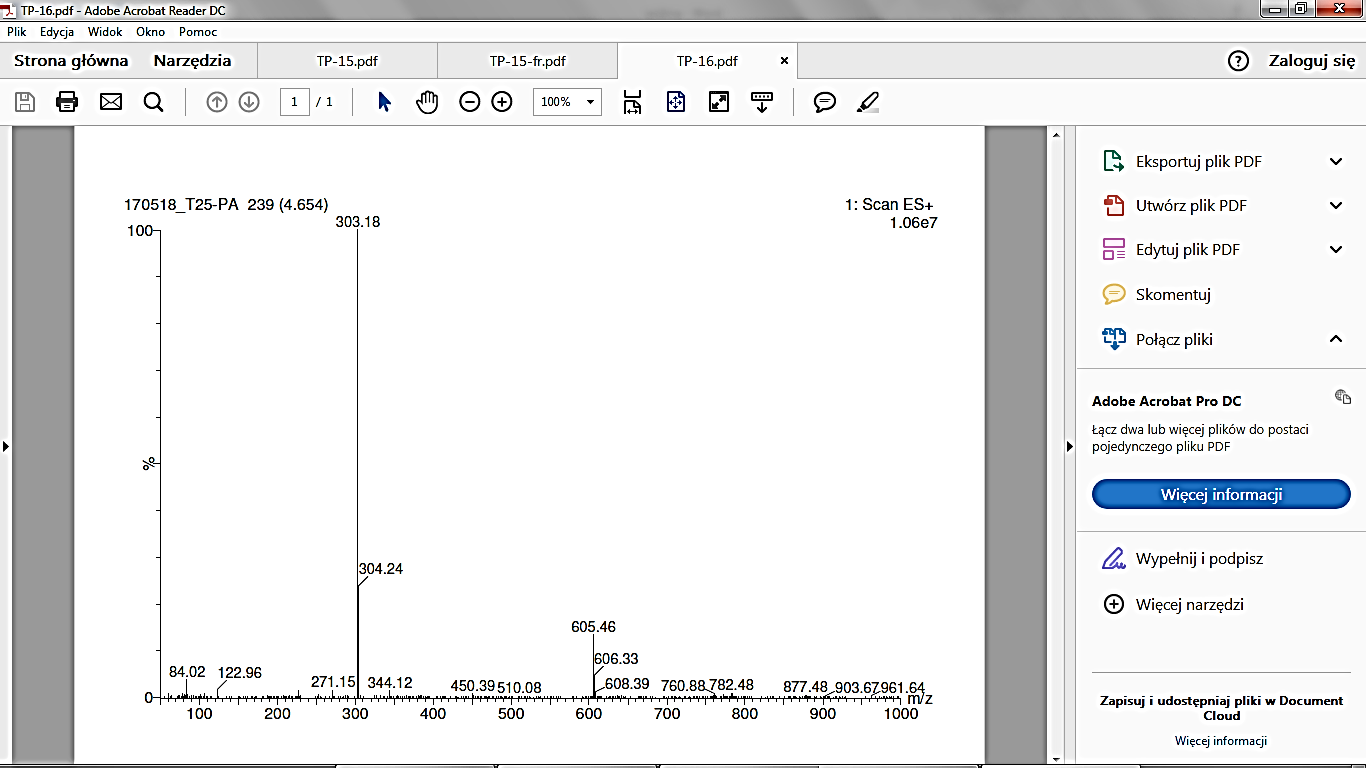


Fig. 53. MS spectrum of TP-16


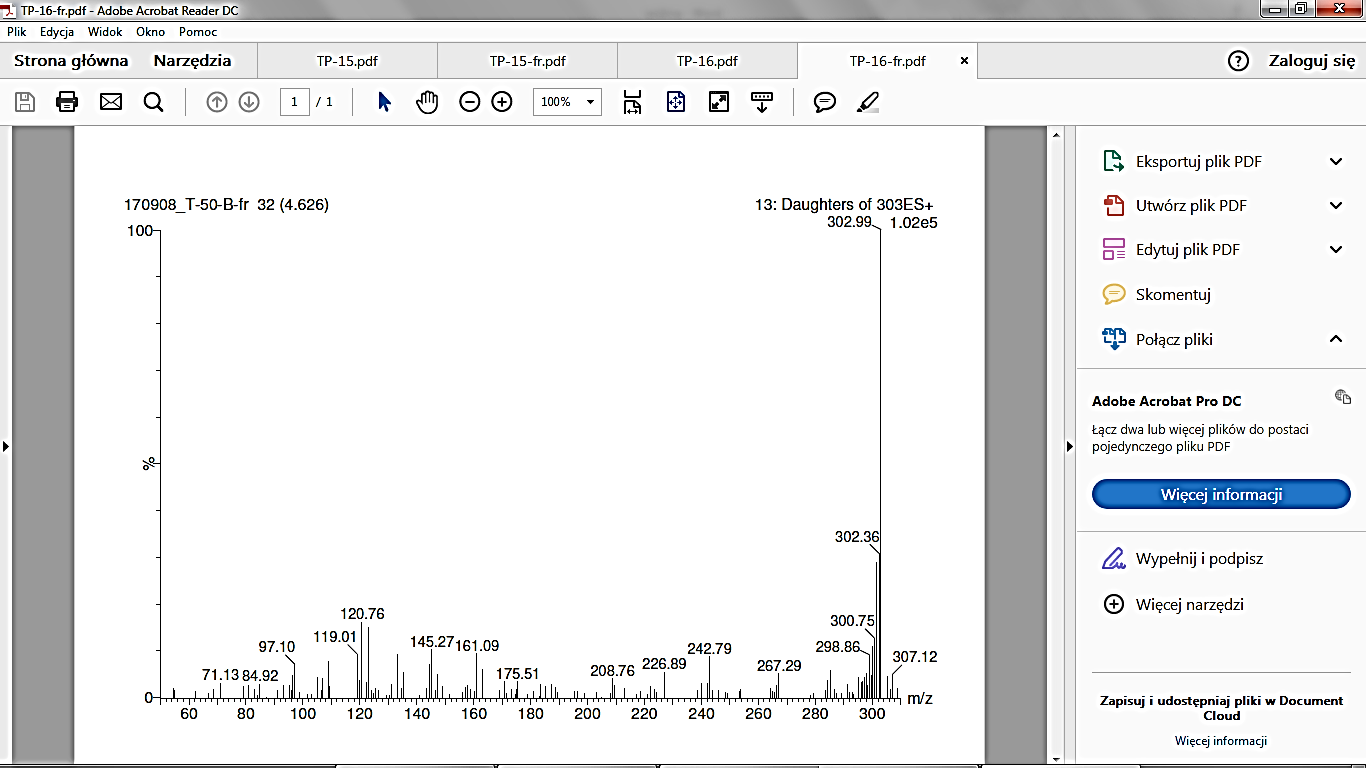


Fig. 54. MS/MS spectrum of TP-17
